# Supplementary material for: Comprehensive Environmental Assessment of Potato as Staple Food Policy in China
Source: Int J Environ Res Public Health. 2019 Jul 29;16(15):2700. doi: 10.3390/ijerph16152700 (PMC6695635; doi:10.3390/ijerph16152700)
Supplement: Supplementary file 1 [file ijerph-16-02700-s001.pdf]

Supplementary material

Comprehensive environmental assessment of potato as staple food policy in China

Bing Gao[a,b], Wei Huang[a,c], Xiaobo Xue[d], Yuanchao Hu[a,b], Yunfeng Huang[e], Lan Wang[a,c], Shengping Ding[a,c], Shenghui Cui[a,b]*

[a] Key Lab of Urban Environment and Health, Institute of Urban Environment, Chinese Academy of Sciences, Xiamen 361021, China

[b] Xiamen Key Lab of Urban Metabolism, Xiamen 361021, China

[c] University of Chinese Academy of Sciences, Beijing 100049, China

[d] Department of Environmental Health Sciences, State University of New York at Albany, 12144, NY

[e] School of Biotechnology Engineering, Jimei University, Xiamen, 361021, PR China

Corresponding author: **Shenghui Cui**

Institute of Urban Environment, Chinese Academy of Sciences, 1799 Jimei Road, Xiamen 361021, China.

Phone: +86-592-6190777; Fax: +86-592-6190977.

E-mail: shcui@iue.ac.cn

Includes 81 pages, and 10 tables and 4 figures.

Chemical N-, P_2O_5- and K_2O-fertilizer and irrigation-water inputs and their use efficiencies for producing potatoes, wheat and rice

Average chemical N inputs for rice and wheat were 209 kg ha^{-1} in 6592 farmers' rice fields, and 210 kg ha^{-1} in 6940 farmers' wheat fields, in Chinese main cereal production areas (Chen et al., 2014). National average chemical P_2O_5 and K_2O inputs for rice and wheat were 86 and 109 kg P_2O_5 ha^{-1}, and 85 and 101 kg K_2O ha^{-1}, respectively (Zhang et al., 2008; Li et al., 2010), which are summarized based on investigations of more than 7000 farmers. Under conventional practices, chemical N-, P_2O_5- and K_2O-fertilizer and irrigation-water use amounts are 167, 112

and 146 kg ha^{-1} and 970 m^3 ha^{-1} for potatoes (Fig. S1). They are 256, 123 and 87 kg ha^{-1} and 1940 m^3 $ha^{-1}$for irrigated winter wheat in the NCP, and 176, 91 and 116 kg $ha^{-1}$and 3410 m^3 ha^{-1} for early rice (Fig. S1).Per unit of area, then, chemical N- and P_2O_5-fertilizer and irrigation-water consumptions for potatoes are lower than those for wheat in the NCP and for early rice, except for the P_2O_5 application amount on early rice; but the chemical K_2O-fertilizer of potatoes higher than that of wheat and rice. Different inputs and yields lead to large differences in the use efficiencies of chemical N-, P_2O_5- and K_2O-fertilizer, and irrigation water per unit of product (Table S1). The national mean partial factor productivities of N, P_2O_5, K_2O (PFP_N,$PFP_{P2O5}$and PFP_{K2O},in kilograms of standard grain per kilogram of N, P_2O_5, and K_2O applied) are 33, 41 (Chen et al., 2014) and 36 kg kg^{-1} N (Table S1), 58, 97 (Zhang et al., 2008; Li et al., 2010) and 58 kg kg^{-1} P_2O_5, 65, 70 (Zhang et al., 2008; Li et al., 2010)and 39 kg kg^{-1} $K_2O$for wheat, rice and potatoes, respectively, in China. And GHG emissions in potato's production significant lower than that of wheat and maize (Kong and Zhu, 2016). We further calculated the PFP_N, PFP_{P2O5}, PFP_{K2O} and IWUE for early rice across China and winter wheat in the NCP (Fig. S1).They were 35.8 kg kg^{-1} N, 57.6 kg kg^{-1} P_2O_5, 39.3 kg kg^{-1} K_2O and 3.2 kg m^{-3} for potatoes, 28.0 kg kg^{-1} N, 55.5 kg kg^{-1} P_2O_5, 81.9 kg kg^{-1} K_2O and 3.9 kg m^{-3} for wheat on the NCP, and 43.7 kg kg^{-1} N, 98.8 kg kg^{-1} P_2O_5, 68.7 kg kg^{-1} K_2O and 2.7 kg m^{-3} for early rice. The PFP_N for potatoes falls into the range between early rice across China and wheat on the NCP. Potatoes have a PFP_{P2O5}similar to wheat, and both of them are significantly lower—by about 40%—than that of early rice. The PFP_{K2O} of potatoes significantly lower—by about 52% and 36%—than that of winter wheat on the NCP and early rice, respectively. The IWUEs of the three crops followed the ranking wheat > potato > rice.

We further analyzed the consumption of chemical N-, P_2O_5-andK_2O-fertilizer and irrigation water, and their use efficiencies, in optimized potato management practices, and compared them with conventional practices (Table S5 and Fig. S2). The results showed that the optimized-management chemical N-, P_2O_5-and K_2O-fertilizer applications and irrigation water use forpotatoeswere161, 107 and 161 kg ha^{-1}, and 850 m^{-3} ha^{-1}, respectively. N and P_2O_5 applications similar to conventional practices, while optimized-management K_2O application increased by 10.3% and irrigation water decreased by about 12.4% relative to conventional practices; however, potatoes had higher optimized-management PFP_N, PFP_{P2O5}, PFP_{K2O} and IWUE values,

increasing by 23.7%, 22.2%, 9.4% and 67.7%, respectively, compared to those of conventional ones, because optimized potato yields increased by 26.9%, with similar or slightly higher inputs, relative to conventional farming practices.

The principle of collecting the data for accounting total GHG of different cropping systems

The principle of collecting soil N_2O and CH_4 emissions was that they must have been measured at least for an entire cropping season in field conditions under local farmers' practices. We defined three types of management as (*i*) the direct conventional treatment in literature, (*ii*) NPK plus straw treatment in long-term field experiment, (*iii*) high N treatment (close to the local fertilization level) in N gradient field tests. Data describing the test site, initial and end time, and the cumulative emissions of N_2O and $CH_4$was recorded, and we simultaneously collected information on fertilizer input, and the application of irrigation (or power use for irrigation), pesticides, fuel, and plastic film, if this was directly reported or indirectly reported as CO_2-eq emissions from fertilizer, irrigation, pesticides, fuel and film in the studies. For the latter situation, the rates of irrigation (or power use for irrigation), fuel consumption, pesticides application and plastic film were calculated by the CO_2-eq emissions divided by the CO_2-eq emission parameters of per unit of material consumption in the articles. However, only a few studies on soil N_2O and CH_4 emissions and GWP of crop systems have simultaneously reported the rates of irrigation, irrigation power or the electric charge of irrigation, fuel consumption and pesticides application (Table S1). The missing values of CO_2-eq emissions from these agronomy managements collected from the associated field results or the studies on the carbon footprint of the same cropping systems.

Electricity consumption has been reported in different forms in the collected literature, e.g., it was reported directly; or CO_2-eq emissions from the consumed electricity was reported, electricity rate was estimated by the CO_2-eq emission caused by irrigation divided by the strength coefficient of carbon used in literature; or the cost of electricity (yuan ha^{-1} $year^{-1}$) was reported, and we estimated electricity rate by the cost divided by the average price of electricity in China (0.5 yuan kwh^{-1}) (Table S4). Also, the electricity consumption estimated by irrigation rate (mm $year^{-1}$) multiplying with the reviewed mean electricity cost of per unit irrigation rate in China (4.3 kwh mm^{-1}) (Table S4). This value fall into the range of 2.1–6.4 kwh mm^{-1} ha^{-1} calculated by the survey data from 366 villages in 11 main groundwater using provinces (Wang

et al., 2012).

Fossil fuel combustion for farm operations was collected in three methods, e.g., the weight of fuel was reported directly (kg ha^{-1} year^{-1}); or estimated fuel rate by the CO_2-eq emission from fuel combustion divided by the per unit fuel CO_2 emission applied in the literature; or calculated by the fuel expenditure on power divided by diesel prices.

Plastic film used for mulching crops to save water was collected by the direct weight of film and paper bags or the weight of CO_2-eq from plastic film divided by CO_2-eq coefficient of plastic film, or estimated by the cost of plastic film dividing by the mean film price (12 yuan kg^{-1}) (Yu et al., 2015).

Calculations of the demand for rice and wheat grain, fertilizer and irrigation-water inputs and GHG emissions under different scenarios

We calculated the demand for rice and wheat grain based on rice and flour consumption and the ratios between rice, flour, and the harvest grain in 2012 (Fig. 2 and Table S9). And we calculated the total chemical N-, P_2O_5- and K_2O-fertilizer applications on rice, wheat and potatoes in 2012 using average inputs (Zhang et al., 2008; Li et al., 2010; Chen et al., 2014) multiplied by each sown area from the Chinese Statistical Yearbook (NBSC, 2013). The demands for chemical N-, P_2O_5- and K_2O-fertilizers for rice and wheat under the BAU scenario were estimated from the predicted rice and wheat consumptions in 2020 divided by the rice and wheat consumptions in 2012, multiplied by the total chemical N, P_2O_5 and K_2O inputs for rice and wheat in 2012, respectively. The demand for chemical N-, P_2O_5- and K_2O-fertilizers for potatoes under the BAU was calculated using the collected per hectare inputs for potatoes multiplied by a target sown area of 6.7 million ha. For other scenarios, we first calculated N, P_2O_5 and K_2O reductions from substituting rice and wheat with potatoes, divided by the PFP_N, PFP_{P2O5}, and PFP_{K2O} values for early rice and winter wheat in the NCP. We then subtracted the reductions in N, P_2O_5 and K_2O from the total inputs for rice, wheat and potatoes under the BAU.

The irrigation water for wheat in 2012 was calculated by taking winter wheat area in the NCP and winter wheat area across China except for NCP multiplied by irrigation intensity, respectively (Table S1 and S2). The same principle was used for estimating irrigation water for early, medium and late rice, and potatoes. We calculated irrigation water for potatoes under the BAU by multiplying the irrigation intensity with the government's target of 6.7 million

sown ha in 2020 (MOA, 2016). Irrigation water for wheat production under the BAU in 2020 was estimated by dividing the demand for wheat in 2020 by the weighted IWUE for wheat, which was estimated by IWUE for winter wheat in the NCP multiply with its sown area plus IWUE for winter wheat across China except for NCP multiply with its sown area, divide by the total sown area of winter wheat across China. The same principle was used for estimating the irrigation water input for rice under the different scenarios in 2020.

The same principle for the calculation of irrigation water consumption was used for estimating GHG emissions from rice, winter wheat and potatoes production across China in 2012 and BAU scenario in 2020. And GHG emissions from rice, winter wheat and potatoes under different substitution scenarios were calculated by the total GHG emission of each crop subtracting the GHG reduction from substituting potatoes for rice and wheat, divided by the GHGI values for early rice across China and winter wheat in the NCP.

Description of the distributions of early rice, winter wheat and potatoes at county-level

The study boundaries follow the geographic boundaries of China, 2411 counties are included and excluded Taiwan, Hong Kong, and Macao because of limited data availability (Wu et al., 2014). We first collected the sowing area of different crops at county scale in 2010 based on the statistical yearbook in 2011. 2225 counties accounted for 92.3% of the total counties within the study boundaries have the data on crop planting, others don't have due to limited statistical yearbook of the prefecture-level city, includes three crops (early rice, winter wheat, and potato). Early rice planting area was estimated through the proportions of early, medium, and late rice based on provincial statistical yearbook 2011 if there is only total rice planting area in some prefecture-level towns. And winter wheat planting area is distinguished by integrating the counted wheat area in prefecture-level cities with the types of wheat (spring and winter wheat) on province level. Then we obtained county levels' early rice, winter wheat, and potatoes systems and their sowing area.

At the same time, we have extracted the image data on paddy fields and upland based on a 30 m × 30 m resolution China's land use map in 2010 (Wu et al., 2014). The spatial patterns and grid numbers of paddy fields and upland at county-level were obtained according to county boundaries data associated with the extracted image data on paddy fields and upland. Then we have received the spatial patterns of the three cropping systems mentioned in above

according to the spatial patterns of paddy fields and upland on county-level associated with county levels' cropping systems and planting area, and given different color layers for various cropping systems (Fig. S1). The principle was winter wheat and potatoes systems completely randomized distributed in upland and early rice system utterly randomized distributed in paddy fields, as a result of no available information on the distribution of different cropping systems at county-level. Thereby, Fig. S1 only represents the possible planting area of early rice, winter wheat and potatoes at county-level.

Description of the error propagation equation of mathematical statistics

The error propagation equation of mathematical statistics (IPCC, 2001) as the following formulae S1 and S2 were used for analyze the uncertainty of the per capita potatoes consumption as vegetable plus staple food, national chemical N-, P_2O_5-, K_2O-fertilizer and irrigation water inputs, and total GHG emissions.

$$U_{total} = \frac{\sqrt{(U_1 \cdot x_1)^2 + (U_2 \cdot x_2)^2 + \cdots + (U_n \cdot x_n)^2}}{x_1 + x_2 + \cdots x_n} \qquad \text{(S1)}$$

where U_{total} is the combined uncorrelated uncertainty in the sum of the quantities (half the 90% confidence interval divided by the total (i.e. mean) and expressed as a percentage); x_i and U_i are the uncertain quantities and the percentage uncertainties associated with them, respectively, including per capita potato-as-vegetable and potato-as-a-staple food consumptions, per unit sown area chemical N-, P_2O_5-, K_2O-fertilizer and irrigation water inputs, soil GHG emissions and indirect CO_2 emissions from agronomic managements and their percentage uncertainties for calculating the required per capita potato-as-vegetable and potato-as-a-staple food consumptions, under the goals of 30% and 50% of PSF consumption in China's urban and rural areas in 2020, national chemical N-, P_2O_5-, K_2O-fertilizer and irrigation water inputs, and total GHG emissions from rice, wheat and potato systems.

$$U_{total} = \sqrt{(U_1^2 + U_2^2 + \cdots + U_n^2)} \qquad \text{(S2)}$$

where U_{total} is the combined uncorrelated uncertainty in the product of the quantities (half the 90% confidence interval divided by the total and expressed as a percentage); U_i are the percentage uncertainties associated with each of the quantities, including percentage uncertainties of the population, urbanization rate, the per capita tuber, rice and flour consumption, the proportion of potatoes to other tubers, the partial factor productivities of chemical N, P_2O_5, K_2O (PFP_N, PFP_{P2O5} and PFP_{K2O}, in kilograms of standard grain per kilogram of N, P_2O_5, and K_2O applied), irrigation-water use efficiencies (IWUE), and GHGI for each crop for calculating the predicted per capita rice, flour and potatoes consumption, national rice, flour and potatoes consumption, chemical N-, P_2O_5-, K_2O-fertilziers and irrigation water inputs, and GHG emissions variation driven by the different proportions of potatoes substituting for rice and flour.

Table S1. CO_2-eq emission from N_2O and CH_4 emissions, irrigation, fuel, pesticides, film and fertilizers input, Chemical N-, P_2O_5- and K_2O fertilizer and irrigation-water inputs for potatoes, wheat in the North China Plain (NCP), and early rice, and yields of the three crops, and partial factor productivities of fertilizers N (PFP_N), P_2O_5 (PFP_{P2O5}), K_2O (PFP_{K2O}) and irrigation water production efficiency (IWUE).

Cropping systems /Site	N_2O emission	CH_4 emission	Irrigation	Fuel	Pesticides	Film	N	P_2O_5	K_2O	Yield	Standard grain*	PFP_N	PFP_{P2O5}	PFP_{K2O}	I[illegible]
	kg CO_2-eq ha^{-1}		mm yr^{-1}/kg CO_2-eq ha^{-1}	kg CO_2-eq ha^{-1}			kg yr^{-1}/kg CO_2-eq ha^{-1}			Mg ha^{-1}		kg kg^{-1} N	kg kg^{-1} P_2O_5	kg kg^{-1} K_2O	k[illegible]
**Potatoes**															
Harbin, Heilongjiang							150/1245	90/136	150/147	34.3	6.9	45.7	76.2	45.7	
Hengshui, Hebei			185/1045	0		712				37.5	7.5				4[illegible]
Tongxin, Ningxia			0/0				97/805	104/156	60/59	16.3	3.3	34.0	31.7	55.0	
Tongxin, Ningxia							173/1432	25/37	110/107	18.5	3.7	21.4	148.0	33.6	
Shuizhong, Liaoning							270/2241	150/227	225/221	51.3	10.3	38.1	68.7	45.8	
Wuchuan, Inner Mongolia	522	-27	0/0				90/747	45/68	60/59	11.9	2.4	26.7	53.3	40.0	
Kunming, Yunnan	656						125/1038	75/113	125/123	14.7	2.9	23.2	38.7	23.2	
Wuchuan, Inner Mongolia	139	-19	0/0				90/747	45/68	60/59	19.3	3.9	43.3	86.7	65.0	
Wuchuan, Inner Mongolia	282		135/551				180/1494								

Wuchuan, Inner Mongolia	91					130/1079	300/453	208/204	31.7	6.3	48.5	21.0	30.3	
Xincheng, Liaoning						270/2241	150/227	225/221	43.2	8.6	31.9	57.3	38.2	
Luancheng, Hebei		138/781	73	0										
Liangshan, Sichuan				145	1425	56/467	56/85	56/55	41.7	8.3	148.2	148.2	148.2	
Hejing, Xinjiang		341/1928	374	147	1995									
Zhangjiakou, Hebei		97/550	150	304	1351				15.0	3.0				3
Lanzhou, Gansu		0/0				90/747	210/317	90/88	25.2	5.0	56.0	24.0	56.0	
Wuchuan, Inner Mongolia		0/0				120/996	120/181	150/147	14.5	2.9	24.2	24.2	19.3	
Wuchuan, Inner Mongolia		0/0				120/996	120//181	150/147	20.1	4.0	33.5	33.5	26.6	
Dingxi, Gansu		247/1395							20.9	4.2				1
Dingxi, Gansu		195/1100				60/498	53/80		26.6	5.3	88.7	100.4		4
Minqin, Gansu		290/1638				192/1594	273/412	90/88	54.2	10.8	56.5	39.7	120.0	3
Tongzhou, Beijing		0/0				303/2515	160/242	178/174	24.4	4.9	16.1	30.5	27.5	
Xinxiang, Henan		104/588				169/1403			6.3	1.3	7.5			1
Wuxi, Chongqing						120/996	60/91	180/176	20.3	4.1	33.8	67.7	22.8	

Chengkou, Chongqing		165/1370	120/181	90/88	19.6	3.9	23.8	32.7	43.3	
Xingxiang, Henan	161/910	233/1934	122/184	117/115	21.5	4.3	18.5	35.2	36.8	2
Guyuan, Ningxia	0/0	225/1868	120/181	0/0	26.3	5.3	23.4	43.8		
Leishan, Guizhou		175/1453	204/308	191/187	34.3	6.9	39.2	33.6	36.1	
Tai'an, Shandong		126/1046	66/100	84/82	32.9	6.6	52.2	99.7	78.6	
Dingxi, Gansu	0/0	180/1494	105/159	90/88	13.6	2.7	15.1	25.9	30.0	
Dalateqi, Inner Mongolia		300/2490	345/521	375/368	25.8	5.2	17.2	15.0	13.9	
Jingtai, Gansu		300/2490	345/521	375/368	35.0	7.0	23.3	20.3	18.7	
Guyuan, Heibei		300/2490	345/521	375/368	32.5	6.5	21.7	18.8	17.3	
Lishui, Zhejiang		80/664	0/0	0/0	16.1	3.2	40.3			
Wuchuan, Inner Mongolia	120/678	225/1868	81/122	203/199	21.4	4.3	19.0	52.8	21.2	3
Wuchuan, Inner Mongolia	180/1017	225/1868	81/122	203/199	26.3	5.3	23.4	64.9	26.1	2
Wuchuan, Inner Mongolia	240/1356	225/1868	81/122	203/199	31.1	6.2	27.6	76.8	30.5	2

Weining, Guizhou	0/0				21.6	4.3				
Dingxi, Gansu	0/0	58/481	83/125	0/0	15.8	3.2	54.5	38.1		
Wuchuan, Inner Mongolia	120/678	150/1245	75/113	270/265	31.4	6.3	41.9	83.7	23.3	5
Wuchuan, Inner Mongolia	120/678	150/1245	75/113	270/265	27.3	5.5	36.4	72.8	20.4	4
Shenyang, Liaoning		78/674			46.5	9.3	119.2			
Wuchuan, Inner Mongolia		143/1187	143/216	227/223	36.8	7.4	51.7	51.7		
Luancheng, Hebei	209/1181	105/872	180/273	130/127	21.4	4.3	40.8	23.8	33.1	2
Yongchun, Fujian		330/2739	165/249	264/259	26.5	5.3	16.1	32.1	20.1	
Qiqihaer, Heilongjiang	0/0	145/1204	92/139	280/274	28.7	5.7	39.6	62.4	20.4	
Xiangyang, Hubei		465/3860	240/362	225/221	18.0	3.6	7.7	15.0	16.0	
Beibei, Chongqing		69/573	56/85	0/0	20.8	4.2	60.3	74.3		
Beibei, Chongqing		69/573	56/85	0/0	19.9	4.0	57.7	71.1		
Wuchuan, Inner Mongolia	0/0	95/789	110/166	150/147	8.7	1.7	18.4	15.9	11.3	
Dingxi, Gansu	0/0	60/498	53/80	75/74	26.6	5.3	88.7	100.4	70.7	

Wuchuan, Inner Mongolia	120/678	169/1403	180/272	248/243	31.6	6.3	37.4	35.1	25.4	5[illegible]
Jinan, Shandong	114/644	113/938	113/171	113/111	35.9	7.2	63.5	63.5	63.7	4[illegible]
Jiaozhou, Shandong	267/1508	225/1868	225/340	225/221	44.5	8.9	39.6	39.6	39.6	3[illegible]
Tai’an, Shandong		150/1245	90/136	210/206	41.4	8.3	55.3	92.2	39.5	
Wuqiao, Hebei	75/424	164/1361	68/103	280/274	19.5	3.9	23.8	57.4	13.9	5[illegible]
Wuqiao, Hebei	90/508	164/1361	68/103	280/274	19.0	3.8	23.2	55.9	13.6	4[illegible]
Wuqiao, Hebei	105/593	164/1361	68/103	280/274	18.0	3.6	22.0	52.9	12.9	3[illegible]
Wuqiao, Hebei	120/678	164/1361	68/103	280/274	17.3	3.5	21.1	50.9	12.5	2[illegible]
Wuchuan, Inner Mongolia		150/1245	75/113	270/265	36.7	7.3	48.9	97.9	27.0	
Wuchuan, Inner Mongolia	0/0	128/1062	45/68	165/162	22.1	4.4	34.5	98.2	26.7	
Wuchuan, Inner Mongolia	135/763	128/1062	45/68	165/162	31.1	6.2	48.6	138.2	37.6	4[illegible]
Wuchuan, Inner Mongolia	270/1525	128/1062	45/68	165/162	33.8	6.8	52.8	150.2	41.2	2[illegible]
Wuchuan, Inner Mongolia	0/0				19.1	3.8				
Wuchuan, Inner Mongolia	0/0	90/747	45/68	60/59	19.2	3.8	42.7	85.3	63.3	
Wuchuan, Inner	0/0	90/747	45/68	60/59	17.1	3.4	38.0	76.0	56.7	

Mongolia										
Wuchuan, Inner Mongolia	0/0	90/747	45/68	60/59	13.9	2.8	30.9	61.8	46.7	
Wuchuan, Inner Mongolia	0/0	90/747	45/68	60/59	16.2	3.2	36.0	72.0	53.3	
Wuchuan, Inner Mongolia	360/2034	225/1868	150/227	270/265	31.9	6.4	28.4	42.5	23.7	1
Wuchuan, Inner Mongolia	180/1017	225/1868	150/227	270/265	34.4	6.9	30.6	45.9	25.6	3
Damaoqi, Inner Mongolia	202/1141	168/1394	54/82	50/49	15.5	3.1	18.5	57.4	62.0	1
Damaoqi, Inner Mongolia	202/1141	168/1394	54/82	50/49	17.0	3.4	20.2	63.0	68.0	1
Damaoqi, Inner Mongolia	202/1141	168/1394	54/82	50/49	14.0	2.8	16.7	51.9	56.0	1
Damaoqi, Inner Mongolia	202/1141	168/1394	54/82	50/49	11.3	2.3	13.5	41.9	46.0	1
Huining, Gansu	0/0	104/863	72/109	0/0	11.8	2.4	22.7	32.8		
Yulin, Shaanxi	457/2582	165/1370	101/153	113/110	36.5	7.3	44.2	72.3	64.6	1
Yangling, Shaanxi	0/0	149/1237	60/91	271/266	25.1	5.0	33.7	83.7	18.4	
Pengyang, Ningxia	0/0	95/789	68/103	75/74	23.1	4.6	48.6	67.9	61.3	

Hunyuan, Shanxi		150/1245	60/91	75/74	32.7	6.5	43.3	108.3	86.7	
Pengyang, Ningxia	0/0				25.9	5.2				
Lanzhou, Gansu	183/1034				36.1	7.2				3
Lanzhou, Gansu	179/1011				30.4	6.1				3
Lanzhou, Gansu	174/983				31.2	6.2				3
Lanzhou, Gansu	171/966				34.2	6.8				4
Changsha, Hunan		248/2058	48/72	161/158	22.3	4.5	18.0	92.9	27.9	
Changsha, Hunan		225/1868	225/340	225/221	33.6	6.7	29.9	29.9	29.8	
Fuzhou, Fujian	0/0	215/1784	146/220		22.1	4.4	20.6	30.3		
Nan’an, Fujian		269/2233	171/258	225/221	22.6	4.5	16.8	26.4	20.0	
Zhangpu, Fujian		225/1868	92/139	225/221	25.2	5.0	22.4	54.8	22.2	
Yunnan province		285/2366	149/225	112/110	19.8	4.0	13.9	26.6	35.7	
Mid-southern Areas of Ningxia		147/1220	84/127	0/0	29.3	5.9	39.9	69.8		
Xiji, Ningxia	0/0	150/1245	90/136	90/88	8.6	1.7	11.5	19.1	18.8	
Luliang, Yunnan	360/2034	288/2390	162/245	180/176	30.1	6.0	20.9	37.2	33.3	1
Yuanzhou, Ningxia		142/1179	52/79	3/3						

Xiji, Ningxia		173/1436	99/149	0/0					
Guyuan, Ningxia		103/855	40/60	0/0					
Southern mountain region, Ningxia		142/1179	66/100	1/1					
Yanchi, Ningxia	0/0	165/1370	81/122	0/0	38.2	7.6	46.3	94.3	
Guyuan, Ningxia	0/0	225/1868	150/227	270/265	27.1	5.4	24.1	36.1	20.0
Guyuan, Ningxia	0/0	108/896			28.9	5.8	53.5		
Dingxi, Gansu	0/0	150/1245	105/159	135/132	14.1	2.8	18.8	26.9	20.7
Zhangxian, Gansu	0/0	120/996	60/91	105/103	35.0	7.0	58.3	116.7	66.7
Yuzhong, Gansu	0/0	150/1245	150/227	75/74	29.9	6.0	39.9	39.9	80.0
Dingxi, Gansu	0/0	179/1486	147/222	82/81	35.3	7.1	38.5	46.9	86.6
Dingxi, Gansu	0/0	104/863	105/159	135/132	14.1	2.8	27.1	26.9	20.7
Dingxi, Gansu	0/0	180/1494	105/159	60/59					
Dingxi, Gansu	0/0	173/1436	90/136	0/0	21.2	4.2	24.5	47.1	
Nanning, Guangxi		160/1328	150/227	360/353	29.2	5.8	36.3	38.7	16.1

Zhongwei, Ningxia					249/2067	95/143	120/118	22.5	4.9	19.7	51.6	40.8	
Weining, Guizhou					240/1992	120/181	270/265	20.6	4.1	17.1	34.2	15.2	
Huining, Gansu					180/1494	90/136	120/118	35.4	7.1	39.4	78.9	59.2	
Zhangye, Gansu	214/1209							40.4	8.1				3
China's average			218										
Shandong province			551					44.2	8.8				
Xiji, Gansu		53											
Chayouhouqi, Inner Mongolia		177						31.2	6.2				
Southern of Ningxia		0											
Guyuan, Ningxia				0									
Hunan province	0/0		215	0									
Wuchuan, Inner Mongolia			564	324									
Zhangye, Gansu			236	240									
Dehong, Yunnan			326	0									

Baotou, Inner Mongolia					362	1140									
Guyuan, Ningxia					515										
Nanan, Fujian						1140									
Nanan, Fujian						998									
Xiji, Ningxia				219											
Guizhou province				0											
Leishan, Guizhou					77										
Qiannan et al., Guizhou				0	45	0									
Kunming, Yunnan				0	105										
Zhoucheng, Shandong				0											
Zhenba, Shaanxi						831									
**Mean ± SE**[#]	338±109	-23±6	97±13/ 538±74	80±35	254±47	725±173	165±7/ 1370±61	110±9/ 166±11	141±11/ 140±11	25.8±1.0	5.2±0.2	35.8±2.3	57.6±3.4	39.3±2.9	3
**Winter wheat in NCP**															
Dongbeiwang, Beijing	150		199/1124				300/2490								
Dongbeiwang,	192	-21	324/1830				300/2490								

Beijing.													
Shangzhuang, Beijing	695	-17	215/1215	138	56	300/2490	160/242	90/88	5.9	19.7	36.9	65.6	2
Yucheng, Shandong	659					210/1743							
Huantai, Shandong	1124		250/1412			270/2241	105/159	105/103					
Huantai, Shandong	442	-26	300/1695			300/2490	120/181	0/0	6.8	22.7	56.7		2
Huantai, Shandong	841		240/1356			270/2241	105/159	60/59	6.1	22.6	58.1	106.7	2
Huantai, Shandong	697	-48				270/2241	105/159	60/59	5.7	21.1	54.3	95.0	
Tai’an, Shandong	659	-21	160/639	278	110	325/2698	150/227	180/176	5.9	18.2	39.3	32.8	3
Tai’an, Shandong	650	-59	160/639	240	113	305/2532	180/272	180/176	6.9	22.6	38.3	38.3	4
Fengqiu, Henan	282					250/2075	75/113	150/147					
Fengqiu, Henan	450					150/1245	75/113	150/147	7.8	52.0	104.0	52.0	
Yongji, Shanxi	702	-20				175/1453	105/159	36/35	5.5	31.4	52.4	152.8	
Yongji, Shanxi	515		216/1220			180/1494	105/159	43/43	6.2	34.4	59.0	144.2	2

Baoding, Hebei	110		240/1356			300/2490	90/136	90/88					
Baoding, Hebei	534					300/2490	150/227	180/176	6.1	20.3	40.7	33.9	
Quzhou, Hebei	370	-57	240/1356	185	46		90/136	60/59					
Quzhou, Hebei	351		200/1130			300/2490	90/136	60/59	7.8	26.0	86.7	130.0	3
Wuqiao, Hebei	164	-14	175/990	163	50	150/1245	60/91	124/122					
Quzhou, Hebei	440	-27	194/1096	92	86	300/2490	120/181	100/98	5.4	18.0	45.0	54.0	2
Quzhou, Hebei	294	-19	180/1017			200/1660	120/181	60/59	3.3	16.5	27.5	55.0	1
Luancheng, Hebei	520	-23				400/3320	65/98	0/0					
Henshui, Hebei	498					300/2490	150/227	90/88					
Luancheng, Hebei	414	-35				400/3320	65/98	0/0					
Baoding, Hebei	1157					329/2731							
Wangdu, Hebei	969	-26				165/1370	60/91	60/59					
Haidian, Beijing	609	-19				105/872							
Xinxiang, Henan	316		240/651	349	11	225/1868	150/227	90/88					
Wuqiao, Hebei	557		289/1633	500	10	247/2050	180/273	76/74					
Luancheng, Hebei			262/1555	155	82	245/2034	151/228	39/38	7.2	29.4	47.7	184.6	2

Luancheng, Hebei	225/781	159	124	225/1868	113/171	225/221	6.8	30.2	60.2	30.2	3[illegible]
Handan, Hebei			140				5.7				
Shangqiu, Henan		585	14	309/2565	127/192	50/49	6.5	21.0	51.2	130.0	
Xingzhou, Shanxi	33/142	688	29	107/888	108/163	107/105	3.6	33.6	33.3	33.6	
Guanzhong, Shaanxi	238/1350	149	5	180/1489	170/257	37/36	6.1	33.9	35.9	164.9	2[illegible]
Luancheng, Hebei	223/1260						6.3				2[illegible]
Shandong province				369/3063							
Hongdong, Shanxi				388/3220	137/207	42/41	6.1	15.7	44.5	145.2	
Dongbeiwang, Beijing	263/1486			300/2490	180/272	0/0					
Dongbeiwang, Beijing	315/1780			300/2490	180/272	142/139	6.0	20.0	33.3	42.3	1[illegible]
Huang-Huai-Hai Plain	300/1695										
Dongbeiwang, Beijing				300/2490	103/156	0/0	5.2	17.3	50.5		
North China				281/2332			5.5	18.1			
North China Plain				325/2698			6.7	20.6			

Longkou, Shandong	300/1695	240/1992	105/159	135/132	8.1	33.8	77.1	60.0	[illegible]
Huimin, Shandong		369/3063	120/181	90/88	6.4	17.3	53.3	71.1	
Luancheng, Hebei	290/1638	512/4250	169/255	0/0	7.0	13.7	41.4		[illegible]
Wuqiao, Hebei	300/1695	261/2166	135/204	113/111	8.1	31.0	60.0	71.7	[illegible]
Tai'an, Shandong	102/576	240/1992	113/171	113/111	9.0	37.5	79.6	79.6	[illegible]
Yanzhou, Shandong	59/333	240/1992	113/171	113/111	7.9	32.9	69.9	69.9	
Tai'an, Shandong	120/678	210/1743	150/227	113/111	9.2	43.8	61.3	81.4	[illegible]
Yanzhou, Shandong	120/678	210/1743	105/159	105/103	8.0	38.1	76.2	76.2	[illegible]
Dezhou, Shandong	150/847	180/1494	150/227	150/147	8.0	44.4	53.3	53.3	[illegible]
Yanzhou, Shandong	180/1017	300/2490	105/159	105/103	7.6	25.3	72.4	72.4	[illegible]
Tai'an, Shandong	180/1017	210/1743	120/181	105/103	7.5	35.7	62.5	71.4	[illegible]
Tai'an, Shandong	180/1017	210/1743	120/181	105/103	7.6	36.2	63.3	72.3	[illegible]
Xinji, Hebei	180/1017	270/2241	150/227	75/74	7.8	28.9	52.0	104.0	[illegible]
Dezhou, Shandong	270/1525	240/1992	150/227	75/74	8.5	35.4	56.7	113.3	[illegible]

Jiaozuo, Henan	190/1073	257/2133	137/207	53/51	7.1	27.6	51.8	134.0	[illegible]
Kaifeng, Henan	0/0	225/1868	90/136		4.3	19.1	47.8		
Quzhou, Hebei	245/1384	300/2490			6.7	22.3			[illegible]
Wuqiao, Hebei	225/1271	250/2075	78/118	0/0	7.8	31.2	100.0		[illegible]
Yucheng, Shandong	220/1243	300/2490	300/453	75/74	7.3	24.3	24.3	97.3	[illegible]
Zibo, Shandong	0/0	210/1743	150/227	150/147	5.6	26.7	37.3	37.3	
Tai’an, Shandong	0/0	240/1992	113/171	113/111	4.9	20.4	43.4	43.4	
Tai’an, Shandong	180/1017	300/2490	105/159	75/74	6.6	29.3	62.9	88.0	[illegible]
Tai’an, Shandong	180/1017	210/1743	150/227	113/111	8.9	42.4	59.3	78.8	[illegible]
Yanzhou, Shandong	120/678	210/1743	150/227	113/111	8.0	38.1	53.3	70.8	[illegible]
Yanzhou, Shandong	99/559	251/2083	158/239	145/142	8.8	35.1	55.7	60.7	[illegible]
Yanzhou, Shandong	73/418	240/1992	150/227	150/147	8.4	35.0	56.0	56.0	
Hongdong, Shanxi		150/1245	60/91	0/0	4.6	20.4	76.7		
Baoding, Hebei		285/2366	120/181	150/147	7.4	32.9	61.7	49.3	

Quzhou, Hebei		304/2523			6.4	22.3			
Wuqiao, Hebei	150/847	215/1785	203/307	150/147	8.1	37.7	39.9	54.0	[illegible]
Huanghuaihai Plain		207/1718	115/174	61/60	6.2	33.9	62.5	101.6	
Northern Plateau		130/1079	87/131	16/16	4.3	48.1	53.5	268.8	
Wuqiao, Hebei	300/1695	300/2490	90/159	90/88	6.0	20.0	66.7	66.7	[illegible]
Huaiyuan, Anhui		270/2241	120/159	120/118	5.9	26.2	49.2	49.2	
Taian, Shandong	173/977	210/1743	90/136	75/74	7.3	32.4	81.1	97.3	[illegible]
Juxi, Anhui		225/1868	105/159	105/103	4.7	20.9	44.8	44.8	
Wuqiao, Hebei	253/1429	158/1311	139/210	113/111	7.4	32.9	53.2	65.5	[illegible]
Wuqiao, Hebei	225/1271	240/1992	130/210	120/118	9.0	37.5	69.2	75.0	[illegible]
Quzhou, Hebei	170/960	240/1992	80/121	90/88	6.7	27.9	83.8	74.4	[illegible]
Linfen, Shanxi	225/1271	157/1307	135/204	75/74	5.5	35.0	40.7	76.0	[illegible]
Guanzhong, Shaanxi		199/1652	142/214	37/36	5.9	26.2	41.5	159.5	
Nanpi, Hebei					6.9				

Fengqiu, Henan							250/2075	150/227	105/103	5.2	23.1	34.7	49.5	
**Mean ± SE**	530±49	-29±4	194±11/ 1421±310	289±49	62±12	0	256±8/ 2122±63	123±5/ 186±7	87±6/ 86±6	6.6±0.2	28.0±1.1	55.5±2.1	81.9±5.9	3
**Early rice**														
Yingtan, Jiangxi	464						104/863	180/272	90/88					
Hangzhou, Zhejiang			454/2566				214/1776	45/68	68/67	5.8	27.1	128.9	85.3	1
Hangzhou, Zhejiang			528/2985				214/1776	45/68	68/67	5.8	27.1	128.9	85.3	1
Hangzhou, Zhejiang			550/3106				214/1776	45/68	68/67	5.7	26.6	126.7	83.8	1
Wuhan, Hubei							275/2283	138/204	275/270	8.3	30.2	60.1	30.1	
Beibei, Chongqiong		5763					125/1038	60/91	75/74					
Jinxian, Jiangxi	229	4790					150/1425	75/113	135/132	7.8	52.0	104.0	57.8	
Jinxian, Jiangxi	304	2633					150/1425	75/113	135/132	8.0	53.3	106.7	59.3	
Jinxian, Jiangxi	793	2038					150/1425	75/113	135/132	8.0	53.3	106.7	59.3	
Taihe, Jiangxi		3558					179/1486							
Taoyuan, Hunan	54	6343					81/672	90/136	85/83	6.3	77.8	70.0	74.1	
Taoyuan, Hunan	18						81/672	90/136	85/83	7.0	86.4	77.8	82.4	

Taoyuan, Hunan		698			81/672	90/136	85/83	6.1		75.3	67.8	67.8
Wangcheng, Hunan	356	4360			75/623	45/68	120/118	5.5		73.3	122.2	45.8
Changsha, Hunan	268	1921			150/1245	90/136	90/88	5.3		35.3	58.9	58.9
Changsha, Hunan	440	3812			165/1370	75/113	105/103	7.1		43.0	94.7	67.6
Changsha, Hunan	136	11328			98/813	76/115	90/88/					
Changsha, Hunan	158	3906			137/1137	137/207	137/134					
Ningxiang, Hunan	18	6308			144/1195	45/68	53/52					
Ningxiang, Hunan	158	3488			240/1992	120/181	120/118	9.5		39.6	79.2	79.2
Liuyang, Hunan	60	5140			150/1245	30/45	60/59	6.2		41.3	206.7	103.3
Wuxue, Hubei		12100		39	140/1162	70/106	140/137	5.9		42.1	84.3	42.1
Baiyun, Guangdong	94	3825			150/1245	45/68	128/125	4.9		32.7	108.9	38.3
Changsha, Hunan	86	8496			150/1245	90/136	113/111	5.9		39.3	65.6	45.4
Jinxian, Jiangxi	292	2450			150/1245	75/113	135/132	7.8		52.0	104.0	57.8
Changsha, Hunan	318	2970			150/1245	120/181	240/235					
Changsha, Hunan	367	1273			150/1245	120/181	240/235					

Ningxiang, Hunan	64	6706		401	38	218/1809	68/103	74/73	6.0	27.5	88.2	81.1	
Chongming, Shanghai	312	8806	52/294	293	486	278/2307	74/111	108/106	9.6	34.5	129.7	88.9	
Jiangjin, Chongqing	158	2978	116/554	199	17	225/1868	120/181	75/74	7.7	34.2	64.2	102.7	6
Taoyuan, Hunan			169/955						5.8				3
Jinxian, Jiangxi			350/1977	125	68	90/747	45/68	75/74	8.8	97.8	195.6	117.3	4
Hunan province			218/931	344	201	179/1486	180/272	41/40					
Pingluo, Ningxia			404/2285	504	50	330/2739	75/113		8.8	26.7	117.3		2
Yangzhou/Suzhou, Jiangsu			284/1605	595	235	269/2224	58/88	55/54	7.6	28.3	131.0	138.2	2
Rugao, Jiangsu			350/1980	376	219								
Average in China					75								
Yanting, Sichuan				147	135								
Taihu lake region			125/705	125	135								
Nanjing, Jiangsu			733/4141			156/1295	180/272	60/59	7.4	47.4	41.1	123.3	1
Gaoyou, Jiangsu			706/3989			310/2573			9.4	30.3			1
Nancang, Jiangxi			82/463			180/1494	68/103	150/147	6.1	33.9	89.7	40.7	7

Nancang, Jiangxi	70/395	180/1494	68/103	150/147	6.3	35.0	92.6	42.0	9
Yingtan, Jiangxi	164/926								
Yujiang, Jiangxi	286/1616				5.0				1
Nancang, Jiangxi		210/1743	90/136	180/176	6.7	31.9	74.4	37.2	
Wuhan,Hubei	394/2226								
Shanghai city	349/1972				7.9				2
Jiangsu province	306/1729				7.8				2
Zhejiang province	362/2045				6.9				1
Anhui province	323/1825				6.1				1
Jiangxi province	350/1977				6.3				1
Hubei province	326/1842				8.4				2
Hunan province	321/1814				6.9				2
Jiujiang, Jiangxi	248/1401	225/1867	125/189	158/155	5.2	23.1	41.6	32.9	2
Qianjiang, Hubei		310/2573	75/113	120/118					
Wangcheng,		150/1425	39/59						

Hunan															
Chengdu, Sichuan							180/1494	60/91	120/118	8.9		49.4	148.3	74.2	
Beibei, Chongqing							125/1038	60/91	90/88						
Hangzhou, Zhejiang							210/1743	105/159	135/132	8.3		39.5	79.0	61.5	
Hangzhou, Zhejiang							210/1743	105/159	135/132	8.6		41.0	81.9	63.7	
Yanting, Sichuan							150/1245	150/227	124/122	6.2		41.3	41.3	50.0	
Yujiang, Jiangxi			282/1591				184/1527	91/137	186/182	8.1		44.0	89.0	43.5	2[illegible]
Jianyang, Sichuan			630/3358				150/1245	72/109	135/132	8.8		58.7	122.2	65.2	1[illegible]
Jingmen, Hubei			539/3045				169/1403	90/136	90/88	8.3		49.1	92.2	92.2	1[illegible]
Nanning, Guangxi							195/1619	54/82	113/111	7.2		36.9	133.3	63.7	
Lianyungang, Jiangsu			658/3717				225/1868			7.0		31.1			1[illegible]
Shiyan, Hubei			256/1446							6.0					2[illegible]
Wuxue,Hubei			294/1661												
Wuxue,Hubei			315/1780												
**Mean ± SE**	234±39	4820±607	341±30/ 1927±204	311±52	142±38		176±8/ 1464±69	87±5/ 129±8	116±7/ 111±7	7.1±1.3		43.7±2.7	98.8±6.0	68.7±4.3	2[illegible]

*Standard yield of grain = Yield of potato × 0.2 (Li et al., 2006).

#Mean± Stand error.

Table S2. CO_2-eq emission from N_2O and CH_4 emissions, fertilizers input, power for irrigation, fuel in farm operations, pesticide application, and film in winter wheat across China except for wheat on the NCP, middle-season rice, and late rice production.

Cropping systems /Site	N_2O emission	CH_4 emission	Fertilizer			Irrigation	Fuel	Pesticide	Film	References
			N	P_2O_5	K_2O					
kg CO_2-eq ha^{-1}										
Winter wheat across China except for NCP										
Yanting, Sichuan	1897		4150			0				Zhou et al., 2013
Yanting, Sichuan	2578	5	1245	181	41	0				Jiang, 2005
Yanting, Sichuan	1224	5	1245	181	41	0				Jiang, 2005
Suining, Sichuan	736		2324			0		89		Zeng et al., 2012
Jiangjin, Chongqing	691	239	1494	181	44	0	199	30		Su, 2016
Jingtang, Sichuan	2321	382				0				Sun, 2007
Yanting, Sichuan	2891		1245	109	44	0				Jiang et al., 2006
Nanjing, Jiangsu	3016		1660	68	132	0				Zou et al., 2005b
Changzhou, Jiangsu			1947	118	0			88		Wang et al., 2009
Qiyang, Hunan	185		2485	181	117	0				Huang et al., 2011
Jiading, Shanghai	1422	178	1901	106	0	0				Huang, 2007
Changshu, Jiangsu	489	20	1743	136	88	0				Zhang et al., 2012d
Changshu, Jiangsu	2121	220	1494	136	176	0				Ma et al., 2013
Suzhou, Jiangsu	3793		1585	43	32	0				Yao et al., 2010
Wuxi, Jiangsu	1452		1494	113	113	0				Yao et al., 2010
Jiangdu, Jiangsu	1030		2075	118	76	0				Yao et al., 2010
Yixing, Jiangsu	1463		1992			0				Huang et al., 2011c
Yangzhou, Suzhou, Jiangsu			2631	125	81	0	364	47		Yan, 2015

Wenxi, Shanxi			1245	227	147	0			Gao et al., 2015b
Beibei, Chongqing		-12	1121	91	59				Xiong, 2013
Yangtze River Delta	716	-13	1868	113	74	0			Yao et al., 2013
Kunshan, Jiangsu	1103		1868			0			Peng et al., 2013
Yanjiang, Jiangsu			1992	136	88	713	270	60	Xue et al., 2015
Chengdu, Sichuan	2259		1359	40	31	0			Gao et al., 2013
Nanhu, Hubei	1442		1868	181	103	0			Liang et al., 2010
Chongming, Shanghai	277	-298	1868			0			Zhang et al., 2015a
Changshu, Jiangsu	609	-5	1743	136	88	0			Zhang et al., 2015c
Chengdu, Sichuan	832	124	1494	113	74	349	828	36	Yang et al., 2015a,b
Luoyang, Henan			1121	136	74	0			Li et al., 2006a
Pingluo, Ningxia			2484	113	15	571	495	42	Zeng, 2013
Xianyang, Shaanxi			1353	135	102	36	92	10	Wang, 2008
Chengcheng, Shaanxi			996	136	0	0			Jia, 2012
Chengcheng, Shaanxi			996	136	0	734			Jia, 2012
Yili, Xinjiang			1743			2219			Xu et al., 2011
Awati, Xinjiang			2179	147	44	2219			Tang et al., 2015
Shaanxi province			1622	192	35	0			Zhao et al., 2013
Wuwei, Gansu			3030	344	0	2797			Yu et al., 2012
Tongwei, Gansu			1245	181	0	0			Lan et al., 2016
Qingyang, Gansu			1992	151	0	0			Zhang, 2017
The Loess Plateau						0			Wei et al., 2017
Anningqu, Xinjiang						1525			Feng et al., 2016
Yangling, Shaanxi			934	170	110	0			Zhang et al., 2017
Yangling, Shaanxi			1826	136	0	0			Hu et al., 2014
Weibei, Shaanxi			1643	186	32	0			Zhao et al., 2016
Guanzhong, Shaanxi			1489	257	36	1350	149	5	Liu et al., 2017
**Mean±SE**	1502±200	70±50	1757±93	146±9	58±8	291±104	342±96	45±11	

Medium rice									
Hailun, Heilongjiang	167	723	792						Liang et al., 2004
Hailun, Heilongjiang	179	620	792						Yue et al., 2005
Sanjiang, Heilongjiang		3213	1568						Xie et al., 2010
Sanjiang, Heilongjiang	623	2371	498						Hao, 2005
Anqing, Heilongjiang	229	628	1494			2083			Chang et al., 2010
Sanjiang, Heilongjiang	796	5200	1245						Chen et al., 2013
Sanjiang, Heilongjiang	875	7669	498						Wang et al., 2008
Shenyang, Liaoning		975	1568						Xie et al., 2010
Shenyang, Liaoning	167	723	792						Liang et al., 2004
Sanjiang, Heilongjiang	627		498						Chen, 2007
Anqing, Heilongjiang	426		872						Wang and Zhang, 2015
Beibei, Chongqing	2104	5763	1043	91	88				Zhang, 2011
Taihu Lake region	230	4367							Yang, 2013b
Beibei, Chongqing		7167	1121	91	59				Xiong, 2013
Beibei, Chongqing	2732	5990	2075						Liu, 2013
Yanting, Sichuan	1484		1245	227	122				Jiang, 2005
Jingtang, Sichuan	1526	10275	1583						Sun, 2007
Yingtan, Jiangxi	464		859						Xiong et al., 2002
Xishuangbanna, Yunnan	1673	4520	1245						Yang, 2007
Chongming, Shanghai	312	8806	2303			290	292	128	Cao et al., 2014
Nanjing, Jiangsu	18	1070	1660	91	117				Zhang, 2013a
Yinchuan, Ningxia			2743			2250	593	50	Zeng et al., 2012
Jiangjin, Chongqing	407	5435	1868			653	199	17	Su, 2016
Pingluo, Ningxia			2742	113	28	2285	594	50	Zeng, 2013
Hunan province			1612	149	124	300	212	48	Lu et al., 2015
Pingluo, Ningxia						2285	632	50	Zeng, 2013

Wuzhong, Ningxia						9380			Zhu, 2003
Fujing, Heilongjiang						4687			Fu, 2000
Jingmen, Hubei						3237			Cheng et al., 2006a
Jingmen, Hubei						6758			Cheng et al., 2006b
Anqing, Heilongjiang						4201			Wei, 2010
Chengdu, Sichuan						5078			Sun, 2010
Wuxue,Hubei						1828			Yao, 2012
Haerbin, Heilongjiang						3209			Zhu, 2012
Baoqing, Heilongjiang						4213			Sun, 2011
Nanning, Guangxi						7605			Liu, 2012
Yongji, Jilin						1744			Wang, 2012
Songnei Plain						3290			Huang et al., 2015
Shuangyashan,Heilongjiang						2384		64	Hu and Liu, 2012
Songyuan and Baicheng, Jilin						892	220	41	Xu, 2011
Heilongjiang Province							274		Xu et al., 2012
Yanting, Sichuan							147	135	Li, 2006
Suzhou, Jiangsu						3314	164	135	Li, 2009
Nongken, Heilongjiang								48	Hu et al., 2008
Chongming, Shanghai						1352	68	39	Dong et al., 2014
Tai Lake Region						705	125	135	Yang, 2013
Hangzhou, Zhejiang								107	Chen et al., 2015
Chengdu, Sichuan						782	581	36	Yang et al., 2015a
Ningxiang, Hunan							337	39	Xue, 2015
Yugan, Jiangxi							152	208	Yan, 2015
**Mean±SE**	711±156	4195±727	1363±132	127±22	90±16	2992±465	306±51	78±13	
Late rice									

Yingtan, Jiangxi	122		2291	290	88	Xiong et al., 2002
Nanjing, Jiangsu	43	990	1660	91	118	Zhang, 2013a
Chengdu, Sichuan	262		1245	170	59	Xiong, 2006
Jingtang, Sichuan	2384	3313				Sun, 2007
Yanting, Sichuan	1132		1245	136	139	Jiang et al., 2006
Chengdu, Sichuan	685		1245	242	147	Yu et al., 2008
Yanting, Sichuan	1131	5695	2075	136	35	Zhou et al., 2015
Yanting, Sichuan	2579	7069	1245	181	40	Jiang, 2005
Yanting, Sichuan	2115	6500	1245	181	0	Jiang, 2005
Beibei, Chongqing	1179	2229	1038	91	74	Liu, 2013
Nanjing, Jiangsu	1577	1183	2515	53	52	Zou et al., 2005a
Nanjing, Jiangsu	2079		2490	68	88	Zou et al., 2005b
Jiading, Shanghai	891	3713	2283	106	0	Huang, 2007
Changshu, Jiangsu	224	9850	1992	181	118	Zhang et al., 2012d
Changshu, Jiangsu	244	6374	2490	136	118	Ma et al., 2013
Yixing, Jiangsu	259		1992			Huang et al., 2011c
Kunshan, Jiangsu	447		2490			Peng et al., 2013
Chengdu, Sichuan	-80		774	23	18	Gao et al., 2013
Nanhu, Hubei	730		1743	113	118	Liang et al., 2010
Suzhou, Jiangsu	2763		1585	50	75	Yao et al., 2010
Wuxi, Jiangsu	937		2075	0	0	Yao et al., 2010
Jiangdu, Jiangsu	656		2075	136	88	Yao et al., 2010
Ningxiang, Hunan	41	13316	1195	68	51	Bai et al., 2010
Yangtze River Delta	534	9353	2075	106	69	Yao et al., 2013
Ningxiang, Hunan	40	13155	909	68	51	Zhang et al., 2013a
Liuyang, Hunan	65	6542	1370	45	59	Kong et al., 2013
Wuxue, Hubei		15000	1162	106	137	Li et al., 2013
Baiyun, Guangdong	178	6700	1245	68	125	Yi et al., 2014

Changsha, Hunan	331	2655	1061	47	54				Qin et al., 2014
Jinxian, Jiangxi	431	3378	1494	113	147				Cheng et al., 2014
Suzhou, Jiangsu			1774	34	41	3314	164	91	Li, 2009
Changzhou, Jiangsu			2922	160	0			88	Wang et al., 2009
Taihu Lake region	241	3075	1445			715	21	30	Yang, 2013b
Jixian, Jiangxi	424	6917	1494	113	147				Shang et al., 2015
Wangcheng, Hunan	417	26823	1494	68	118				Qin et al., 2006
Changsha, Hunan	234	7680	1345	60	132				Shi et al., 2011b
Changsha, Hunan	498	1226	1494	68	110				Qin, 2011
Changsha, Hunan	60	4701	1434	408	265				Peng et al., 2015
Taoyuan, Hunan	70	13485	844	0	151				Shang et al., 2011
Chongming, Shanghai	322	16853	2490						Zhang et al., 2015a
Changshu, Jiangsu	332	2793	1992	181	118				Zhang et al., 2015c
Chengdu, Sichuan	161	3238	1494	136	88	571	905	36	Yang et al., 2015a, b
Jiangjin, Chongqing	379		1868	181	74		199	6	Su, 2016
Yujiang, Jiangxi						1267	413	326	Xiao et al., 2006
Yanjiang, Jiangsu			2490	181	118	713	296	219	Xue et al., 2015
Hunan province			1622	92	76	300	212	48	Lu et al., 2015
Yangzou/Suzhou, Jiangsu			2233	88	54	1605	468	235	Yan, 2015
Liling, Hunan								99	Li et al., 2001
Qianjiang, Hubei			1524	120	24			87	Li et al., 2001
Hunan, province						538	96		Lu et al., 2015
South China							250		Xu et al., 2012
Jiaungsu province							227		Xu et al., 2012
Xiangyin, Hunan								54	Cheng et al., 2011
**Mean±SE**	678±119	7279±1105	1701±78	117±12	85±53	1128±438	296±107	110±44	

Table S3. Irrigation-water intensity, plant area and average yield of early, middle-season and late rice in China.

Period	Site	Irrigation water (m-3 ha-1)	Plant area (× 10³ha-1)*	Average yield (Mg ha-1)	Irrigation water production efficiency (kg·m-3)	
Early rice	Hangzhou, Zhejiang	4542				Cheng, 2001
	Hangzhou, Zhejiang	5284				Cheng, 2001
	Hangzhou, Zhejiang	5497				Cheng, 2001
	Taoyuan, Hunan	3070				Xie et al., 2001
	Jinxian, Jiangxi	2030				Li et al., 2009c
	Wujiang, Jiangsu	4060				Li, 2009
	Hunan province	1750				Huang et al., 2011
	Gaoyou, Jiangsu	7060				Xue, 2013
	Shanghai city	3490				Li et al., 2011a
	Jiangsu province	3060				Li et al., 2011a
	Zhejiang province	3620				Li et al., 2011a
	Anhui province	3230				Li et al., 2011a
	Jiangxi province	3500				Li et al., 2011a
	Hubei province	3260				Li et al., 2011a
	Hunan province	3210				Li et al., 2011a
	Jiujiang, Jiangxi	2480				Zhang et al., 2011b
	Chongming, Shanghai	520				Cao et al., 2014
	Yinchuan, Ningxia	4040				Zeng et al., 2012
	Jingmen, Hubei	11700				Cheng et al., 2006a
	Jingmen, Hubei	10800				Cheng et al., 2006a
	Jingmen, Hubei	5390				Cheng et al., 2006b
	Lianyungang, Jiangsu	6580				Shao et al., 2003

	Shiyan, Hubei	2560				Wang and Liu, 2001
	Chongming, Shanghai	2430				Dong et al., 2014
	Wenjiang, Sichuan	10820				Zhang, 2006
	Yizheng, Jiangsu	9580				Wang, 2003
	Wuxue,Hubei	3150				Yao, 2011
	Wuxue,Hubei	2940				Yao, 2011
	Jianyang, Sichuan	6300				Zhang et al., 2012a
	Yujiang, Jiangxi	2817				Zhao et al., 2007
	Yujiang, Jiangxi	2860				Xiao et al., 2006
	Wuhan,Hubei	3940				Luo et al., 2009
	Nancang, Jiangxi	820				Huang, 2014
	Nancang, Jiangxi	700				Huang, 2014
	Yingtan, Jiangxi	1640				Chen et al., 2000
	Nanjing, Jiangsu	9580				Qian et al., 2003
	Nanjing, Jiangsu	7330				Chen et al., 2011<br>Guo et al., 2010
	Guilin, Guangxi	1304				Lv et al., 2011
	Mean± SE	4393±2929				
	Across China		5765	5.8	1.3±0.1	
Medium rice	Wujiang, Jiangsu	14000				Li, 2009
	Nanning, Guangxi	7640				Liu, 2012
	Nanning, Guangxi	12660				Liu, 2012
	Qingtongxia, Ningxia	13500				Li et al., 2005
	Baoqing, Heilongjiang	10002				Sun, 2011
	Chengdu, Sichuan	8450				Sun, 2010
	Anqing, Heilongjiang	6992				Wei, 2010

Fujing, Heilongjiang	7800	Fu, 2000
Gannan, Heilingjiang	14190	Wang et al., 2007
Yancheng, Jiangsu	12965	Huang et al., 2003
Cangping, Beijing	2800	Zhang et al., 2005
Cangping, Beijing	4978	Zhao, 2004
Cangping, Beijing	2138	Cui et al., 2008
Pingluo, Ningxia	20117	Liu et al., 2005
Yujiang, Jiangxi	10284	Zhao et al., 2007
Anqing, Heilongjiang	6000	Chang et al. , 2010
Yongji, Jilin	4135	Wang, 2012
Wuhan, Hubei	3900	Ke, 2010
Northeast China	7448	Ji et al., 2008a
Northeast China	5847	Ji et al., 2008a
Northeast China	5046	Ji et al., 2008a
Shuangyashan, Heilongjiang	4529	Hu and Liu, 2012
Northern of Hubei	9580	Liu et al., 2016
Nanjing, Jiangsu	14890	Huang, 2004
Nanjing, Jiangsu	8260	Guo et al., 2009
Haerbin, Heilongjiang	5340	Zhu, 2012
Jingmen, Hubei	3044	Lv et al., 2011
Wuxue, Hubei	2740	Liu, 2011
Yangzhou, Jiangsu	5565	Xue, 2013
Lianyungang, Jiangsu	5080	Xue, 2013
Taiyuan, Shanxi	11180	Wang, 2015b
Songnen Plain	4980	Huang et al., 2015

	Anqing, Heilongjiang	4246				Zhuang, 2015
	Mean ± SE	7889±752				
	Across China		18019	7.4	0.9±0.1	
Late rice	Nanjing, Jiangsu	5850				Hao et al., 2015
	Nanjing, Jiangsu	6367				Hao et al., 2015
	Chongming, Shanghai	2425				Dong et al., 2014
	Yongzhou, Hunan	1599				Liang et al., 2000
	Suzhou, Jiangsu	7040				Yin, 2012
	Yujiang, Jiangxi	7333				Zhao et al., 2007
	Guilin, Jiangxi	3193				Lv et al., 2011
	Jinxian, Jiangxi	3540				Li, 2009
	Hezhou, Guangxi	3120				Chen, 2013
	Pingluo, Ningxia	4044				Zeng, 2013
	Fengyang, Anhui	6660				Xiao et al., 2012
	Kunshan, Jiangsu	7118				Liu et al., 2014
	Mean ± SE	4587±598				
	Across China		6354	5.9	1.3±0.1	
	Mean of total rice[#]				1.1±0.1	

※Plant area of different rice from Chinese Agricultural Statistical Yearbook (MOA, 2015).

[#]Mean irrigation water use efficiency of total rice = (Early rice yield × early rice sown area + medium rice yield × medium rice sown area + late rice yield × late rice sown area)/Total sown area of early, medium, and late rice.

Table S4. Irrigation rate, power consumption, power used per unit of irrigation rate and electricity charges in the Chinese main crop systems in different regions.

Site	Crop systems	Irrigation rate (mm)	Power consumption (kWh)	Power used per unit of irrigation rate ($kWh\ mm^{-1}$)	Electricity charge ($yuan\ kWh^{-1}$)	Reference

Shouguang, Shandong	Greenhouse vegetable	1496	4400	2.94		Gao et al., 2009
Shouguang, Shandong	Greenhouse vegetable	1134	2482	2.45		Fan, 2014
Tai'an, Shandong	Wheat-maize system	240	726	3.03	0.50	Wang, 2013
Tai'an, Shandong	Wheat-maize system	160	384	2.40		Tian, 2014
Luancheng, Hebei	Wheat-maize system	425	1910	4.50		Liang et al., 2009
Hebei, province	/	675	3054	4.52	0.40	Wang, 2010
Luancheng, Hebei	Wheat-maize system	300	867	2.89		Yang, 2015a
Wuqiao, Hebei	Wheat-maize system	300	592	1.97		Wang, 2015
Quzhou, Hebei	Wheat-maize system	278	3233	11.6		Gao et al., 2015
Beijing, Shangzhuang	Wheat-maize system	215	1385	6.44		Huang et al., 2013
Quzhou, Hebei	Wheat-maize system	240	1892	8.26		Cao, 2015
Suzhou, Jiangsu	Rcie-rapeseed system	1400	5859	4.19	0.45	Li, 2009
Jinxian, Jiangxi	Double rice system	700	2112	3.02	0.80	Li, 2009
Jinzhong, Shaanxi	Spring maize system	140	500	3.57		Duan et al., 2014
Northeast China	Maize system	300	900	3.00		Chang et al., 2010
Yinchuan, Ningxia	Maize system	275	519	1.89		Zeng, 2013
Zhangye, Gansu	Greenhouse vegetable	1206	3400	2.82		Zhao et al., 2006
Wuwei, Gansu	Maize system	390	3068	7.87		Wu, 2014
Rugao, Jiangsu	Rice-wheat system	/	/	/	0.45	Xue et al., 2015
**Mean± SE**				4.3±0.2	0.50±0.07	

Table S5. CO_2-eq emission from N_2O and CH_4 emissions, and chemical N-, P_2O_5- and K_2O fertilizer (kg CO_2-eq ha^{-1}), Chemical N-, P_2O_5- and K_2O fertilizer and irrigation-water application, yields of potatoes, partial factor productivity of fertilizer N (PFP_N), P_2O_5 (PFP_{P2O5}) and K_2O (PFP_{K2O}), and irrigation-water production efficiency (IWUE) under optimized potato management practices in China.

Site	N_2O	CH_4	N	P_2O_5	K_2O	Irrigation water mm/kg CO_2-eq ha^{-1}	Yield Mg ha^{-1}	Standard grain※ Mg ha^{-1}	PFP_N kg kg^{-1} N	PFP_{P2O5} kg kg^{-1} P_2O_5	PFP_{K2O} kg kg^{-1} K_2O	IWUE kg m^{-3}	Reference
	kg CO_2-eq ha^{-1}		kg ha^{-1}/kg CO_2-eq ha^{-1}										
Wuchuan, Inner Mongolia	291	-42	90/747	45/68	60/59	0/0	9.6	1.9	21.1	42.2	31.7		Gao, 2016
Wuchuan, Inner Mongolia	182	-22	90/747	45/68	60/59	0/0							Gao, 2016
Kunming, Yunnan	337		68/564	75/113	125/123		13.6	2.7	39.7	36.0	21.6		Zhou et al., 2017
Wuchuan, Inner Mongolia	118	-19	90/747	45/68	60/59	0/0	16.2	3.2	35.6	71.1	53.3		Wang, 2015a
Wuchuan, Inner Mongolia	268		90/747			135/763							Wan et al., 2016
Wuchuan, Inner Mongolia	91		60/498	48/72	108/106		31.7	6.3	105.0	131.3	58.3		Shu et al., 2017
Xuanwei, Yunnan			141/1170	105/159	199/195		31.8	6.4	45.4	61.0	32.2		Kong et al., 2004
Taixing, Jiangsu			84/697	45/68	135/132		19.7	3.9	46.4	86.7	28.9		Sun, 2005
Haiyuan, Ningxia			78/647	48/72	96/94		29.5	5.9	75.6	122.9	61.5		Li et al., 2006

Haiyuan, Ningxia	78/647	48/72	96/94		13.7	2.7	34.6	56.3	28.1		Li et al., 2006
Wuchuan, Inner Mongolia	120/996	120/181	150/147		23.1	4.6	38.3	38.3	30.7		Wang et al., 2013a
Wuxi, Chongqing	120/996	60/91	180/176		21.4	4.3	35.8	71.7	23.9		Lv et al., 2010
Chengkou, Chongqing	165/1370	120/181	90/88		21.5	4.3	26.1	35.8	47.8		Lv et al., 2010
Xinxiang, Henan	233/1934	122/184	117/115	119/672	20.4	4.1	17.6	33.6	35.0	3.4	Huang et al., 2010
Tai'an, Shandong	118/979	58/88	105/103		35.6	7.1	60.2	122.4	67.6		Liu et al., 2011b
Tai'an, Shandong	150/1245	90/136	210/206		47.5	9.5	63.3	105.5	45.2		Gao, 2014
Dalateqi, Inner Mongolia	180/1494	180//272	225/221		24.5	4.9	27.2	27.2	21.8		Zhang et al., 2005
Jingtai, Gansu	180/1494	180/272	225/221		33.0	6.6	36.7	36.7	29.3		Zhang et al., 2005
Guyuan, Hebei	180/1494	180/272	225/221		31.5	6.3	35.0	35.0	28.0		Zhang et al., 2005
Dingxi, Gansu	60/498	53/80	75/74		44.8	9.0	150.0	169.8	120.0		Qin et al., 2011
Wuchuan, Inner Mongolia	150/1245	81/122	203/199	180/1017	25.5	5.1	34.0	63.0	25.1	2.8	Chen et al., 2012
North of Yinshan, Inner Mongolia	90/747	90/136		135/763	42.5	8.5	94.4	94.4		6.3	Jing et al., 2012
Xiangyang, Hubei	108/896	99/149	162/159		20.4	4.1	38.0	41.4	25.3		Yang, 2012
Wuqiao, Hebei	164/1361	68/103	280/274	120/678	24.9	5.0	30.5	73.5	17.9	4.2	Wang et al., 2013b
Wuchuan, Inner Mongolia	97/805	110/166	264/259	0/0	15.8	3.2	33.0	29.1	12.1		Li et al., 2013
Wuchuan, Inner Mongolia	165/1370	180/272	248/243	120/678	35.4	7.1	43.0	39.4	28.6	5.9	Qin et al., 2013
Wuwei, Gansu	135/1121	135/204	180/176	0/0	54.1	10.1	74.8	74.8	56.1		Song et al., 2013
Pengyang, Ningxia	95/789	68/103	75/74	0/0	29.8	6.0	63.2	88.2	80.0		Li and Hou, 2015
Qizhou, Shanxi	150/1245	101/153			30.1	6.0	40.0	59.4			Wen et al., 2016
Changsha, Hunan	248/2017	78/118	161/158		27.2	5.4	21.8	69.2	33.5		Lin et al., 2012

Wuchuan, Inner Mongolia	233/1934	150/227	265/260		20.6	4.1	17.6	27.3	15.5		Yue et al., 2013
Lichuan, Hubei	113/938	75/113	120/118		23.9	4.8	42.5	64.0	40.0		Wang et al., 2016b
Middle and southern Ningxia	147/1220	84/129	0/0		38.5	7.7	52.4	91.7			Jia et al., 2012
Wuchuan, Inner Mongolia	62/515	69/104		0/0	19.7	4.0	64.5	58.0			Zhao et al., 2005
Luancheng, Hebei	150/1245	105/159	130/127	132/746	26.7	5.3	35.3	50.5	40.8	4.0	Wang et al., 2005a
Lanzhou, Gansu	83/689	5076	0/0	0/0	15.4	3.1	37.3	62.0			Wang et al., 2005b
Yongchun, Fujian	330/2739	157/237	223/219		30.3	6.1	18.5	38.9	27.4		Chen, 2007
Chengde, Hebei	85/706	29/44	114/112	383/2164	27.0	5.4	63.5	186.2	47.4	1.4	Li et al., 2007
Gaolan, Gansu	185/1536	36/54	0/0	0/0	39.0	7.8	42.2	216.7			Tian et al., 2007
Lishui, Zhejiang	120/996	80/121	180/176		23.3	4.7	39.2	58.8	26.1		Ma and Guo, 2007
Minqin, Gansu	192/1594	273/412	108/106	250/1412	66.3	13.3	69.3	48.7	123.1	5.3	Hou et al., 2008
Dingxi, Gansu	179/1486	147/222	150/147		36.3	7.3	40.8	49.7	48.7		Chen et al., 2008
Liupanshui, Guizhou	105/872	65/98	120/118		33.0	6.6	62.9	101.5	55.0		Ji, 2008b
Minqin, Gansu	192/1594			262/4844	58.3	11.7	60.9			4.5	Wang et al., 2009a
Damaoqi, Inner Mongolia	99/822	54/82	50/49	202/1141	37.0	7.4	74.7	137.0	148.0	3.7	Wang et al., 2009b
Dingxi, Gansu	179/1486	147/222	150/147	90/508	34.2	6.8	38.0	46.3	45.3	7.6	Dou et al., 2009
Hongmen, Henan	169/1403			85/539	11.7	2.3	13.6			2.7	Li et al., 2009a
Luliang, Yunnan	288/2390	122/184	180/176	180/1017	36.6	7.3	25.3	59.8	40.6	4.1	Li et al., 2015
Mountain region of Southern Ningxia	300/2490	200/302	200/196	50/282	21.0	4.2	14.0	21.0	21.0	8.4	Liao, 2009
Pengyang, Ningxia				0/0	39.5	7.9					Zhang et al., 2009a
Pengyang, Ningxia				0/0	47.7	9.5					Zhang et al., 2009a
Ya’an, Sichuan	150/1245	45/68	135/132		28.2	5.6	37.3	124.4	41.5		Li et al., 2009b

Qujing, Yunnan	150/1245	150/227	256/251		34.8	7.0	46.7	46.7	27.3		Cui et al., 2010
Dingxi, Gansu	150/1245	105/159	135/132	0/0	20.7	4.1	27.3	39.0	30.4		Gao et al., 2010
Jianping, Liaoning	105/872	180/272	130/127		34.5	6.9	65.7	38.3	53.1		Liu et al., 2010a
Dingxi, Gansu	180/1494	75/113	75/74		31.1	6.2	34.4	82.7	82.7		Su et al., 2010
Dingxi, Gansu	150/1245	120/181	165/162	45/254	49.8	10.0	66.7	83.3	60.6		Liu et al., 2010b
Huidong, Guangdong	195/1619	92/139	287/281		46.6	9.3	51.3	101.1	32.4		Chen et al., 2010
Dingxi, Gansu	58/481	83/125	0/0	18/102	19.2	3.8	65.5	45.8			Zhang et al., 2011a
Xishui, Guizhou	180/1494	120181	360/353		22.5	4.5	25.0	37.5	12.5		Deng et al., 2011
Qiqihaer, Heilongjiang	145/1204	95/43	140/137		35.6	7.1	49.0	74.7	50.7		Ma et al., 2011
Leishan, Guizhou	240/1992	180/272	270/265		45.1	9.0	37.5	50.0	33.3		Yang, 2011
Leishan, Guizhou	175/1453	204/308	191/187		44.8	9.0	51.4	44.1	47.1		Yang, 2011
Dingxi, Gansu	180/1494	105/159	90/88		16.6	3.3	18.3	31.4	36.7		Tan et al., 2011
Guyuan, Ningxia	63/523	72/109	90/88	0/0	29.5	5.9	93.7	81.9	65.6		Zhou et al., 2011
Xiapu, Fujian	201/1668	41/62	324/318		34.1	6.8	33.8	165.9	21.0		Huang et al., 2012
Enping, Guangdong	195/1619	180/272	240/235		30.9	6.2	31.8	34.4	25.8		Tan et al., 2012
Wuchuan, Inner Mongolia	150/1245	75/113	270/265		42.3	8.5	56.7	113.3	31.5		Zhang et al., 2012b
Taiyuan, Shanxi	135/1121	79/119	150/147	0/0	35.1	7.0	51.9	88.6	46.7		Feng et al., 2012
Wuming, Guangxi	192/1594	192/290	192/188		25.3	5.1	26.6	26.6	26.6		Tang et al., 2012
Wuchuan, Inner Mongolia				56/316	22.0	4.4				7.9	Shen et al., 2012
Jinan, Shandong	113/938	113171	113/111	89/564	36.4	7.3	64.6	64.6	64.6	8.2	Dong et al., 2013
Jurong, Jiangsu	180/1494	120/181	230/225		25.9	5.2	28.9	43.3	22.6		Lei and Wang, 2013
Wuchuan, Inner	135/1121	143/216	132/129		41.9	8.4	62.2	58.7	63.6		Liang et al., 2013

Mongolia											
Dingxi, Gansu	150/1245	105/159	135/132	0/0	21.1	4.2	28.0	40.0	31.1		Tang et al., 2013
Shenyang, Liaoning	180/1494	144/217	264/259		61.8	12.4	68.9	86.1	47.0		Li et al., 2013
Wuchuan, Inner Mongolia	150/1245	75/113	270/265		36.9	7.4	49.3	98.7	27.4		Yang et al., 2013
Wuchuan, Inner Mongolia	128/1062	45/68	165/162	135/763	35.0	7.0	54.7	155.6	42.4	5.2	Zhang et al., 2013a
Wuchuan, Inner Mongolia	150/1245	60/91	150/147	0/0	29.3	5.9	39.3	98.3	39.3		Chen et al., 2013
Jiaozhou, Shandong	112/930	63/95	183/179	101/571	44.6	8.9			48.6	8.9	Zhang et al., 2013c
Dingxi, Gansu	90/747	90/136	120/118	0/0	16.7	3.3	36.7	36.7	27.5		Zhang et al., 2013b
Guyuan, Ningxia	225/1868	150/227	270/265	0/0	35.0	7.0	31.1	46.7	25.9		Mai et al., 2014
Haerbin, Heilongjiang	180/1494	75/113	180/176		38.1	7.6	42.2	101.3	42.2		Wang et al., 2014
Wuchuan, Inner Mongolia	150/1245	75/113	270/265	120678	39.0	7.8	52.0	104.0	28.9	6.5	Xiao, 2014
Huining, Gansu	104/863	72/109	0/0	0/0	24.7	4.9	47.1	68.1			Xue, et al., 2014
Dingxi, Gansu	104/863	105/159	135/132	0/0	22.1	4.4	42.3	41.9	32.6		Xia et al., 2014
Yanchi, Ningxia	165/1370	81/122	0/0		54.1	10.8	65.5	133.3			Zhao et al., 2014
Boluo, Guangdong	180/1494	135/204	150/147		46.0	9.2	51.1	68.1	61.3		Huang et al., 2014
Taipushiqi, Inner Mongolia		90/136			51.2	10.2		113.3			Li et al., 2014
Luliang, Yunnan	288/2390	162/245	180/176	195/1102	39.7	7.9	27.4	48.8	43.9	4.1	Li et al., 2015
Guyuan, Ningxia	180/1494	90/136	135/132	0/0	27.4	5.5	30.6	61.1	40.7		Liang et al., 2015
Qujing, Yunnan	150/1245	90/136	270/265	0/0	32.7	6.5	43.3	72.2	24.1		Yin et al., 2015a
Qujing, Yunnan	150/1245	90/136	270/265	0/0	12.6	2.5	16.7	27.8	9.3		Yin et al., 2015a

Chayouhongqi, Inner Mongloia			270/2241	180/272	285/279	375/2119	47.9	9.6	35.6	53.3	33.7	2.6	Xing et al., 2015
Tongzhou, Beijing				135/204	0/0	140/791	26.1	5.2		38.5		3.7	Feng et al., 2015
Yulin, Shaanxi			165/1370	101/153	113/111	210/1186	52.3	10.5	63.6	104.0	92.9	5.0	Wang and Zhang, 2015
Cangping, Beijing						318/1797	81.0	16.2				5.1	Wang et al., 2015a
Pengyang, Ningxia			69/573	69/104	75/74	0/0	40.6	8.1	117.4	117.4	108.0		Hou et al., 2016
Chayouzhongqi, Inner Mongolia			300/2490	105/159	165/162	165/932	35.6	7.1	23.7	67.6	43.0	4.3	Duan et al., 2016
Wuchuan, Inner Mongolia			210/1743	90/136	165/162	95/537	43.3	8.7	41.4	96.7	52.7	9.2	Duan et al., 2016
Tongwei, Gansu						0/0	40.8	8.2					Han et al., 2016
Chengde, Hebei			276/2291	120/181	150/147		37.7	7.5	27.2	62.5	50.0		Shang et al., 2016
Eerduo, Inner Mongolia			314/2606	101/153	379/371	128/723	36.7	7.3	23.2	72.3	19.3	5.7	Feng et al., 2016
Dingxi, Gansu			180/1494	150/227	180/176		22.5	4.6	25.6	30.7	25.6		Chen et al., 2016a
Jining, Inner Mongolia			160/1328	110/166	110/108	0/0	19.1	3.8	23.8	34.5	34.5		Mu et al., 2016
Dingxi, Gansu			169/1403	90/136	0/0	0/0	21.2	4.2	24.9	46.7			Wang et al., 2016a
Xiangyang, Hubei			180/1494	90/136	150/146		24.0	4.8	26.7	53.3	32.0		Zhang et al., 2016c
Zhongwei, Ningxia			267/2216	139/210	82/80		30.0	6.0	22.5	43.2	73.2		Wang et al., 2016b
Weining, Guizhou			240/1992	120/181	270/265		27.3	5.5	22.9	45.8	20.4		Zhang et al., 2017
**Mean ± SE**	215±41	-28±7	157±6/1303±50	104±5/157±7	158±8/155±8	81±13/458±76	32.4±1.2	6.5±0.2	44.5±2.3	70.5±3.8	42.3±2.6	5.2±0.4	

※ The same as in Table A.1.

Table S6. Per capita habitual food intake in 1982, 1992, 2002 and 2012 (g cap.$^{-1}$ day^{-1}) (Zhai et al., 2005; NHFPC, 2015).

Item	1982		1992		2002		2012	
	Urban	Rural	Urban	Rural	Urban	Rural	Urban	Rural
Rice	217.0	217.0	223.1	255.8	156.5	226.0	130.8	222.7
Flour	218.0	177.0	165.0	189.1	107.8	147.4	134.7	150.4
Other cereals	24.0	137.0	17.0	40.9	14.4	30.2	15.9	17.6
Potatoes	66.0	228	46.0	108	31.9	55.7	28.4	42.8
Beans	6.1	10.0	2.2	4.0	2.3	4.1	2.9	3.7

Table S7. Proportions of kitchen waste composed of cereals, potatoes and beans (Shi, 2014).

Item	Proportion in kitchen waste (%)	
	Mean	Range
Cereals	18	1-27
Potatoes	5	2-30
Beans	3	1-4

Table S8. Chemical N-, P_2O_5- and K_2O-feitilizer inputs, irrigation-water consumptions and total GHG for rice, wheat, conventionally grown potatoes (Potato-Con), and optimally grown potatoes (Potato-Opt) under different scenarios in 2020, compared with BAU scenario (mean±sd).

Item	Rice	Wheat	Potato-Con	Potato-Opt	$Total_1$*	Relative to 2012†	$Total_2$#	Relative to 2012†
N (Tg)								
2012	6.3±3.2	4.7±1.9	0.9±0.4		11.9±3.7			
BAU	6.0±3.2	5.0±2.2	1.1±0.5		12.1±3.9	0.2±0.05		0.2±0.05
$30S_{0R+100F}$	6.0±3.2	4.7±2.1	1.1±0.5	0.9±0.8	11.8±3.9	-0.1±0.02	11.6±3.9	-0.3±0.07
$30S_{50R+50F}$	5.9±3.1	4.9±2.1	1.1±0.5	0.9±0.8	11.9±3.8	0.0±0.0	11.7±3.8	-0.2±0.05
$30S_{100R+0F}$	5.8±3.0	5.0±2.2	1.1±0.4	0.9±0.8	11.9±3.7	0.0±0.0	11.7±3.8	-0.2±0.05
$50S_{0R+100F}$	6.0±3.2	4.3±1.7	1.4±0.9	1.1±1.2	11.7±3.7	-0.2±0.04	11.5±3.8	-0.4±0.09
$50S_{50R+50F}$	5.7±3.0	4.7±1.9	1.4±0.9	1.1±1.2	11.8±3.6	-0.1±0.02	11.6±3.7	-0.3±0.07
$50S_{100R+0F}$	5.5±2.7	5.0±2.2	1.4±0.9	1.1±1.2	11.9±3.6	0.0±0.0	11.6±3.7	-0.3±0.07
P_2O_5 (Tg)								
2012	2.6±0.3	2.4±0.2	0.6±0.3		5.6±0.6			
BAU	2.5±0.5	2.6±0.6	0.7±0.5		5.8±0.9	0.2±0.02		0.2±0.02
$30S_{0R+100F}$	2.5±0.5	2.5±0.5	0.7±0.5	0.6±0.6	5.7±0.9	0.1±0.01	5.5±1.0	-0.1±0.01
$30S_{50R+50F}$	2.4±0.5	2.5±0.5	0.7±0.5	0.6±0.6	5.7±0.9	0.1±0.02	5.6±1.0	0±0.0
$30S_{100R+0F}$	2.4±0.5	2.6±0.6	0.7±0.5	0.6±0.6	5.7±0.9	0.1±0.01	5.6±1.0	0±0.0
$50S_{0R+100F}$	2.5±0.5	2.2±0.4	0.9±0.5	0.7±0.7	5.6±0.9	0.0±0.00	5.4±1.0	-0.2±0.02
$50S_{50R+50F}$	2.4±0.5	2.4±0.5	0.9±0.6	0.7±0.7	5.7±0.9	0.1±0.01	5.5±1.0	-0.1±0.01
$50S_{100R+0F}$	2.2±0.5	2.6±0.6	0.9±0.6	0.7±0.7	5.7±1.0	0.1±0.01	5.6±1.0	0±0.0
K_2O (Tg)								
2012	3.0±0.5	1.9±0.4	0.8±0.6		5.7±0.9			
BAU	2.9±0.7	2.0±0.6	0.9±0.7		5.9±1.2	0.2±0.03		0.2±0.03

$30S_{0R+100F}$	2.9±0.7	1.9±0.5	0.9±0.7	0.9±1.0	5.8±1.1	0.1±0.01	5.7±1.3	0±0
$30S_{50R+50F}$	2.8±0.7	2.0±0.5	0.9±0.7	0.9±1.0	5.8±1.1	0.1±0.01	5.7±1.3	0±0
$30S_{100R+0F}$	2.8±0.6	2.0±0.6	0.9±0.7	0.9±1.0	5.7±1.1	0.0±0.0	5.7±1.3	0±0
$50S_{0R+100F}$	2.9±0.4	1.8±0.5	1.3±0.9	1.3±1.4	6.0±1.2	0.3±0.04	6.0±1.7	0.3±0.05
$50S_{50R+50F}$	2.7±0.6	1.9±0.5	1.3±0.9	1.3±1.4	5.9±1.2	0.2±0.03	6.0±1.7	0.3±0.05
$50S_{100R+0F}$	2.6±0.6	2.0±0.6	1.3±0.9	1.3±1.4	5.9±1.2	0.2±0.03	5.9±1.6	0.2±0.03
Irrigation water (× 10^9 m^3)								
2012	198.3±81.1	35.9±15.5	5.4±0.7		239.6±82.5			
BAU	189.0±85.0	38.3±18.0	6.5±0.8		233.7±86.8	-5.9±1.5		-5.9±1.5
$30S_{0R+100F}$	189.0±85.0	36.1±16.1	6.5±0.8	3.9±1.6	231.6±86.5	-8.0±2.0	229.0±86.5	-10.6±2.7
$30S_{50R+50F}$	187.1±83.3	37.2±17.0	6.5±0.8	3.9±1.6	230.7±85.0	-8.9±2.2	228.2±85.1	-11.4±2.9
$30S_{100R+0F}$	185.2±81.7	38.3±18.0	6.5±0.8	3.9±1.6	229.9±83.6	-9.7±2.4	227.4±83.6	-12.2±3.1
$50S_{0R+100F}$	189.0±85.0	33.2±13.9	9.3±2.0	5.7±2.6	231.5±86.1	-8.1±2.1	227.9±86.1	-11.7±3.0
$50S_{50R+50F}$	184.5±81.1	35.8±15.8	9.3±2.0	5.7±2.6	229.6±82.7	-10.0±2.5	226.0±82.7	-13.6±3.4
$50S_{100R+0F}$	180.1±77.6	38.3±18.0	9.3±2.0	5.7±2.6	227.7±79.7	-11.9±2.9	224.0±79.7	-15.6±3.9
Total GHG (Gg CO_2-eq)								
2012	304.2±87.1	102.8±40.6	20.1±6.4		427.1±96.3			
BAU	289.9±99.1	109.6±43.7	24.2±7.7		423.7±110.4	-3.4±0.6		-3.4±0.6
$30S_{0R+100F}$	289.9±96.8	103.6±41.2	24.2±7.7	17.7±13.5	417.7±113.9	-9.4±1.7	413.0±114.9	-14.1±2.5
$30S_{50R+50F}$	283.4±94.8	106.6±44.9	24.2±7.7	17.7±13.5	414.2±103.5	-12.9±2.2	409.2±106.4	-17.9±3.1
$30S_{100R+0F}$	276.9±90.7	109.6±47.9	24.2±7.7	17.7±13.5	410.7±102.9	-16.4±2.8	405.4±104.1	-21.7±3.7
$50S_{0R+100F}$	289.9±99.1	95.6±39.4	34.8±12.6	25.5±19.9	420.3±107.4	-6.8±1.2	410.9±108.5	-16.2±2.8
$50S_{50R+50F}$	274.7±85.7	106.1±43.7	34.8±12.6	25.5±19.9	415.6±97.1	-11.5±1.9	406.2±98.3	-20.9±3.5
$50S_{100R+0F}$	259.4±81.0	109.6±47.9	34.8±12.6	25.5±19.9	403.8±94.9	-23.3±3.8	394.5±94.6	-32.6±5.4

[*] $Total_1$ = Rice + wheat + potato-Con (conventionally grown potatoes).

[#] $Total_2$ = Rice + wheat + potato-Opt (optimally grown potatoes).

†Relative to 2012 = Different scenario results minus the values of 2012.

Table S9. Energy supply under the different substitution scenarios in 2020, in comparison with 2012.

Scenario	Energy supply*
	× 10^6 cal
2012	652.5
BAU	/
$30S_{0R+100F}$	655.2
$30S_{50R+50F}$	655.6
$30S_{100R+0F}$	656.8
$50S_{0R+100F}$	659.8
$50S_{50R+50F}$	660.9
$50S_{100R+0F}$	663.1

*Per unit energy supply of rice, wheat and potato is 3.6 × 10^6, 3.9 × 10^6 and 4.5 × 10^6 cal kg^{-1}, respectively (Chen, 2002).

Table S10. Comparison of nutritional components between 35% potato bread and 100% wheat bread, and the nutrition of 35% potato bread (250 g) and 100% wheat bread (250 g) (Sun et al., 2015).

Index	100% wheat bread	35% potato bread	Nutrient Reference Values (g)	Weight (%)*	100% wheat bread (250g)			35% potato bread (250g)		
					Provide nutrition (g)	Ratio (%)#	Nutrient supply index†	Provide nutrition (g)	Ratio (%)	Nutrient supply index
Crude protein (g/100g DW)	13.57±0.02	14.29±0.05	60	7.14	33.9	56.5	0.040	35.7	59.5	0.042
Crude fiber (g/kg DW)	0.27±0.02	0.71±0.01	25	7.14	0.1	0.4	0.0003	0.2	0.8	0.0005
Beta carotene (mg/100g DW)	No detection	No detection			no‡	no		no	no	
VB1 (mg/100g DW)	0.21±0.01	0.26±0.01	1.4	7.14	0.5	37.5	0.027	0.7	46.4	0.033
VB2 (mg/100g DW)	0.09±0.01	0.12±0.01	1.4	7.14	0.2	16.1	0.011	0.3	21.4	0.015
VB3 (mg/100g DW)	2.16±0.01	3.87±0.01			no	no		no	no	
VC (mg/100g DW)	7.82±0.12	35.52±0.52	100	7.14	19.6	19.6	0.014	88.8	88.8	0.063
K (mg/100g DW)	342.73±4.51	956.23±1.52	2000	7.14	858.6	42.8	0.031	2390.6	119.5	0.085
P (mg/100g DW)	125.79±5.21	172.53±2.51	700	7.14	314.5	44.9	0.032	431.3	61.6	0.044
Mg (mg/100g DW)	33.29±0.23	57.54±0.21	300	7.14	83.2	27.7	0.020	143.9	48.0	0.034
Ca (mg/100g DW)	31.21±0.17	56.23±2.31	800	7.14	78.0	9.8	0.007	140.6	17.6	0.013
Te (mg/100g DW)	1.29±0.11	2.15±.05	15	7.14	3.2	21.5	0.015	5.4	35.8	0.026
Zn (mg/100g DW)	0.90±0.02	0.98±0.11	15	7.14	2.3	15.0	0.011	2.5	16.3	0.012
Mn (mg/100g DW)	0.43±0.02	0.52±0.01	3	7.14	1.1	35.8	0.026	1.3	43.3	0.031
Cu (mg/100g DW)	0.12±0.01	0.17±0.01	1.5	7.14	0.3	20.0	0.014	0.4	28.3	0.020
Se (μg/100g DW)	5.96±0.01	6.03±.01	50	7.14	14.9	29.8	0.021	15.1	30.2	0.022
As (mg/100g DW)	0.012±0.001	0.012±.002			no	no		no	no	
Pb (mg/100g DW)	No detection	No detection			no	no		no	no	

Polyphenol oxidase A_{416}	0.079±0.002	0.129±0.012			no	no		no	no
Total phenol (mg/g DW)	1.58±0.03	1.93±0.04			no	no		no	no
Antioxidant activity (μg Trolox/g DW)	1542.18±10.23	1726.01±7.12			no	no		no	no
Reducing sugar (g/100g DW)	2.11±0.02	5.31±0.12			no	no		no	no
Crude fat (g/kg DW)	12.90±0.30	10.00±0.20	60		3.2	5.3		2.5	4.2
Na (mg/100g DW)	8.27±0.21	11.67±0.25	2000		20.7	1.0		29.2	1.5
Total				100			0.269		0.441

*Weight (%) = 1/16 × 100, 16 is the total number of index which have the detected Nutrient Reference Values.

#Ratio (%) = Provide nutrition/ Nutrient Reference Values × 100.

†Nutrient index =Ratio (%)×Weight (%)/10000, which represents the extent of total index close to the Nutrient Reference Values.

‡no data.

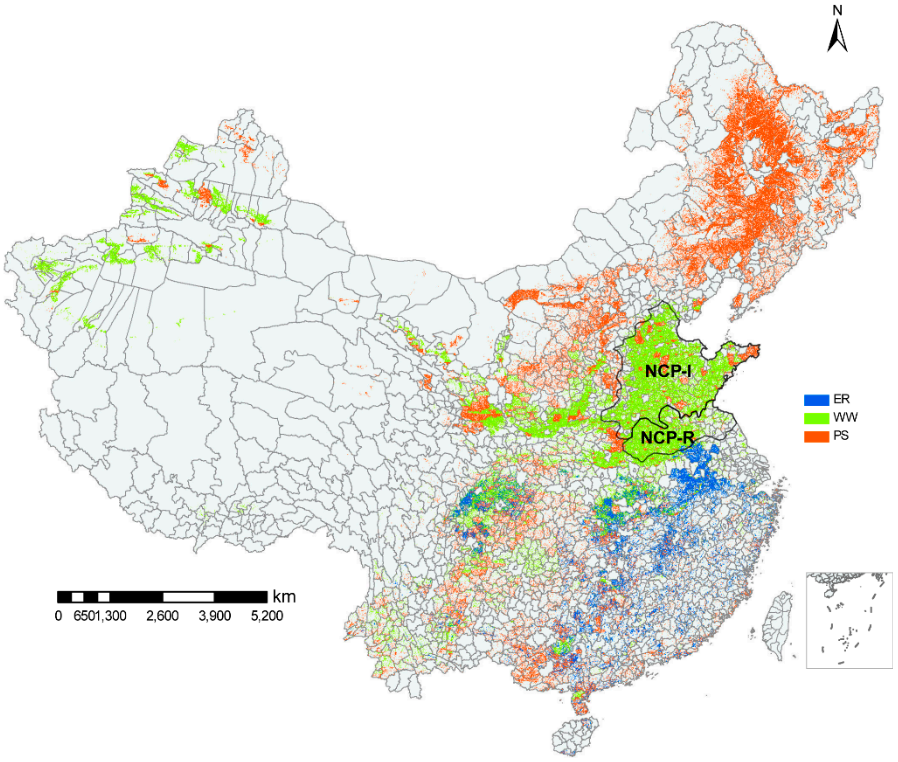

Figure S1. Specific distributions of early rice, winter wheat and potato systems at county-level across China. NCP-I and NCP-R represent the irrigated- and rainfed-winter wheat in the NCP.

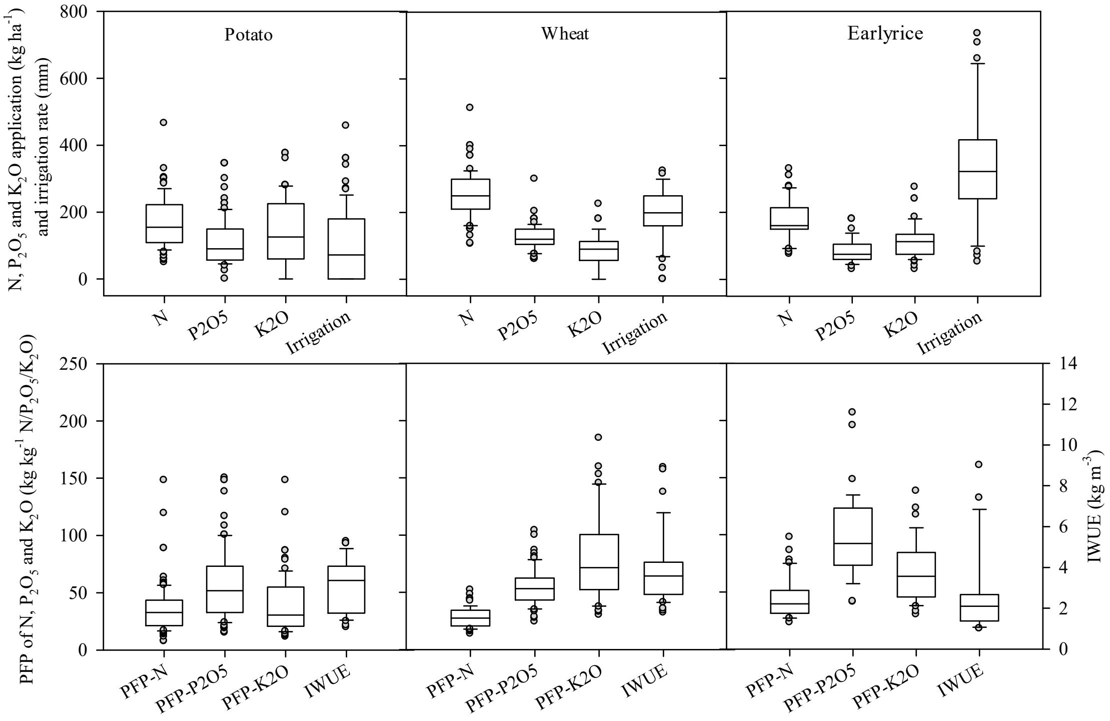

Figure S2. Chemical N-, P_2O_5- and K_2O-fertilizer and irrigation-water consumption and their use efficiencies for potatoes, wheat in the North China Plain, and early rice across China.

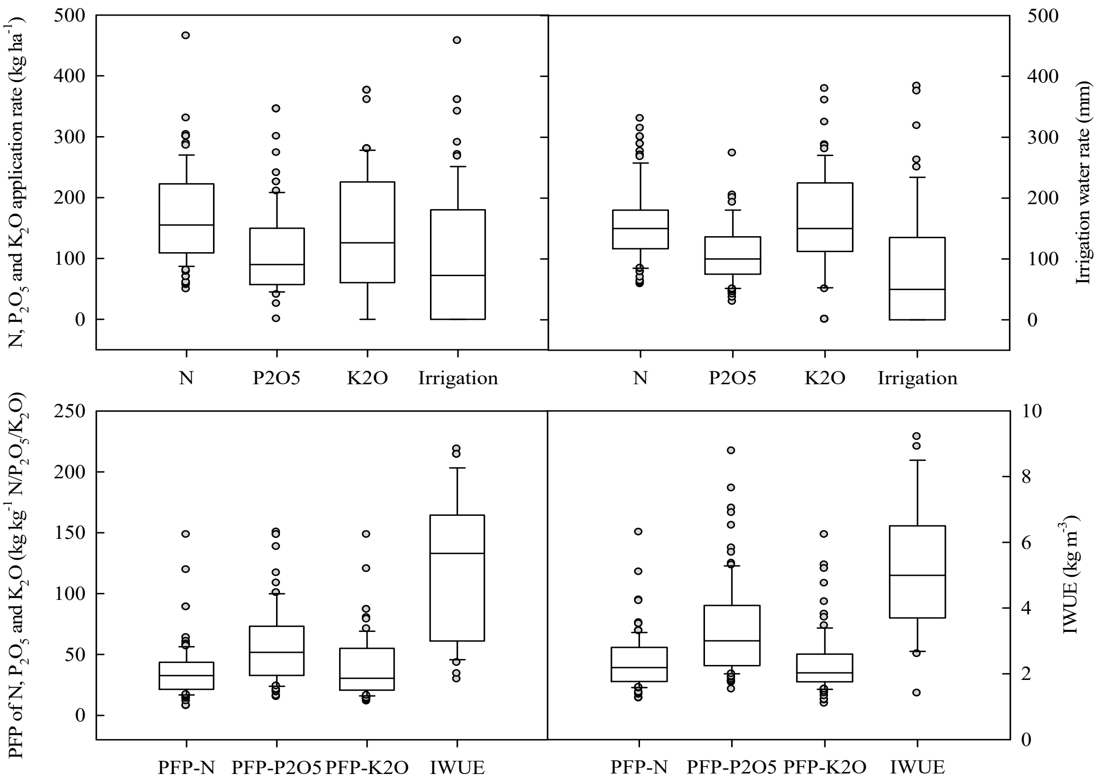

Figure S3. Comparison of chemical N-, P_2O_5-and K_2O-fertilizer and irrigation-water consumption and their use efficiencies, between conventional (left) and optimized (right) potato management practices.

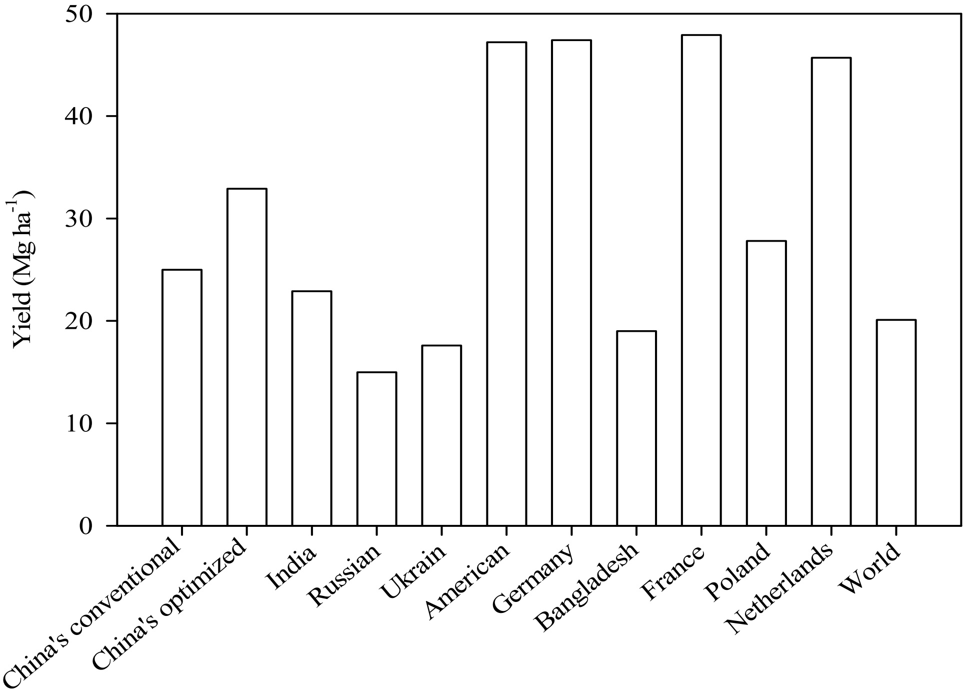

Figure S4. Yields of potato in the top 10 potato-output countries, and average potato yields, worldwide.

References

1. Bai, H.L., Zhang, X.L., Chen, F., Sun, G.F., Hu, Q., Li, Y. 2010.Tillage effects on CH_4 and N_2O emission from double cropping paddy field. Transactions of the CSAE 26(1): 282–289 (in Chinese with English abstract)
2. Cai, J.Z. 2008. Analysis of economic benefit and high yield mulching culture technique of virus-free potato. Fujian Agricultural Science and Technology 1: 75–76. (in Chinese)
3. Cai, Y.J., Ding, W.X., Luo, J.F. 2013. Nitrous oxide emissions from Chinese maize-wheat rotation systems: A 3-yearfield measurement. Atmospheric Environment 65:112–122.
4. Cao, J. 2015. Evaluation of agricultural and environmental performance of combination of organic sources and chemical fertilizer on cropping systems. PhD Thesis, China Agricultural University, Beijing, China.
5. Cao, L.M., Li, M.B., Wang, X.Q., Zhao, Z.P., Pan, X.H. 2014. Life cycle assessment of carbon footprint for rice production in Shanghai. Acta Ecologica Sinica 34(2): 491–499. (in Chinese with English abstract)
6. Chang, S., Zhao, L., Teng, S.M., Zhao, Y.L. 2010. Benefit analysis of mechanization irrigation in arid agriculture areas in Northeast China. Scientific and Technological Extension pp: 66 (in Chinese)
7. Chen, F., 2002. Agroecology. China Agricultural University Press, Beijing, pp. 261 (in Chinese).
8. Chen, F., Zhang, X.J., Wei, Y.Q., Jiang, Z.H., Jia, R.L., Ma, N. 2016a. Nutrient supply to potato under plastic-covered ridge and furrow planting in continuous cropping system in Dingxi city. China Potato Journal 30(1): 20–24. (in Chinese with English abstract)
9. Chen, G.R., Gao, S.M., Zhang, X.Y., Zhang, W., Wang, Y.H. 2008. The effect of potassium application and water supplement in different stages on potato yield and WUE in semiarid area. Agricultural Research in the Arid Areas 26(5): 41–45. (in Chinese with English abstract)
10. Chen, G.R., Wang, L.M., Yang, R.P., Dong, B., Zhang, G.H., Zhang, G.F. 2017. Crop yield and soil fertility affected continuous potato-soybean intercropping systems along the Yellow River. Acta Pratacultura Sinica 26(10): 46–55. (in Chinese with English abstract)
11. Chen, H. 2015. Effect of NPK fertilizer combination on yield and quality of potato. Dissertation of Master Degree, Northwest A&F University, Yangling, Shaanxi, China.
12. Chen, H., Cao, C.F., Kong, L.C., Zhang, C.L., Li, W., Qiao, Y.Q., Du, S.Z., Zhao, Z. 2014. Study on wheat yield stability in Huaibei lime concretion black soil area based on long-term fertilization experiment. Scientia Agricultura Sinica 47(13):2580–2590. (in Chinese with English abstract)
13. Chen, H., Zhang, X.M., Quan, F., Tang, D.F., Cao, X.M. 2010. Influence of different ratios between NPK on yield, economic profit and fertilizer use efficiency of winter potato. China Potato Journal 24(4): 224–229. (in Chinese with English abstract)
14. Chen, H.Y., Meng, M.L., Liang, H.Q., Zhang, J., Wang, Y.H., Wang, Z.X. 2012. Effects of different treatments of irrigation and fertilization on the yield and nitrogen utilization characteristic of potato. Chinese Agricultural Science Bulletin 28(03): 196–201. (in Chinese with English abstract)
15. Chen, J.Z., Chen, M.L., He, Y.Q. 2000. The water balance and productivity of rice under different soil water conditions in red soil area. Journal of Huazhong Agricultural University 19(6): 554–558. (in Chinese with English abstract)
16. Chen, X.P.,Zhang, F.S., Römheld, V., Zhang, F.S., Horlacher, D., Schulz, R., Böning-Zilkens, M., Wang, P., Claupein, W. 2006. Synchronizing N supply from soil and fertilizer and N demand of winter wheat by an improved N_{min} method. Nutrients Cycling in Agroecosystems 74:91–98.
17. Chen, Y., Fan, M.S., Gao, Y. 2013. Effect of micro-ridge with plastic cover and furrow sowing on dry-farming potato yield and soil water content in hill area of Yinshan. Soil and Fertilizer Sciences 5: 71–74. (in Chinese with English abstract)
18. Chen, Y.J. 2013. Effect of different irrigation modes on yield and water use efficiency of rice. Agricultural and Technology 33(10): 108–109. (in Chinese)
19. Chen, Y.X. 2007. The formula application by measuring soil method in winter potato planting. China Potato 21(5): 283–284. (in Chinese)
20. Chen, Z.Y., Guo, X.P., Yao, J.Q. 2011. Water-saving effect of water-catching and controllable irrigation technology of rice. Journal of Hohai University (Natural Sciences) 39(7): 426–430. (in

Chinese with English abstract)
21. Cheng, C., Zeng, Y.J., Yang, X.X., Huang, S., Luo, K., Shi, Q.H., Pan, X.H., Shang, Q.Y. 2015. Effect of different tillage methods on net global warming potential and greenhouse gas intensity in double rice cropping systems. Acta Scientiae Circumstantiae 35(6): 1887–1895. (in Chinese with English abstract)
22. Cheng, J.P., Cao, C.G., Cai, M.L., Wang, J.P., Yuan, B.Z., Wang, J.Z., Zheng, C.J. 2006a. Effects of different irrigation modes on biological characteristics and water use efficiency of paddy rice. Chinese Journal of Applied Ecology 17(10): 1859–1865. (in Chinese with English abstract)
23. Cheng, J.P., Cao, C.G., Cai, M.L., Wang, J.P., Yuan, B.Z., Wang, J.Z., Zheng, C.J. 2006b. Effects of different irrigation modes on the yield and water productivity of rice. Transactions of the CSAE 22(12): 28–33. (in Chinese with English abstract)
24. Cheng, W.D. 2001. Physiological and ecological effects of rice water-saving and high-efficiency cultivation and its influence on yield and quality. Dissertation of Master Degree, Zhejiang University, Hangzhou, China.
25. Chu, P.F., Yu, Z.W., Wang, X.Y., Wu, T.H., Wang, X.Z. 2009. Effects of irrigation amount on grain starch content, starch synthesis activity, and water use efficiency in wheat. Acta Agronomica Sinica 37(8): 1432–1440. (in Chinese with English abstract)
26. Chu, P.F., Wang, D., Zhang, Y.L., Wang, X.Y., Wang, X.Z., Yu, Z.W. 2009. Effects of irrigation stage and amount on water consumption characteristics, grain yield and content of protein components of wheat. Scientia Agricultura Sinica 42(4):1306–1315. (in Chinese with English abstract)
27. Cui, F., Yan, G.X., Zhou, Z.X., Zheng, X.H., Deng, J. 2012. Annual emissions of nitrous oxide and nitric oxide from a wheat–maize cropping system on a silt loam calcareous soil in the North China Plain. Soil Biology & Biochemistry 48: 10–19.
28. Cui, H.Q., Wan, P. 2007. The present situation and development countermeasures of potato mechanization in southern Ningxia region. Mechanization In Rural & Pastoral Areas 4: 46–47. (in Chinese)
29. Cui, X.J., Xue, C.Y., Yang, X.G., Yang, J., Zhang, Q.P., Wang, H.Q., Bouwman, B.A.M. 2008. Field evapotranspiration characteristics and water use efficiency of aerobic rice under different water treatments. Transactions of the CSAE 24(4): 49–54. (in Chinese with English abstract)
30. Cui, X.K., Chen ,J.L., Wang, M.Y., Yang, G.C., Zheng, H.Y. 2010. Effect of fertilization and plant density on potato yield. China Potato Journal 6: 360–363.(in Chinese with English abstract)
31. Cui, Z.L. 2005. Optimization of the nitrogen fertilization management for winter wheat-summer maize rotation system in the North China Plain from field to regional scale. PhD Thesis, China Agricultural University, Beijing, China.
32. Cui, Z.L., Yue, S.C., Wang, G.L., Zhang, F.S., Chen, X.P. 2013. In-season root-zone N management for mitigating greenhouse gas emission and reactive N losses in intensive wheat production. Environment Science and Technology 47: 6015–6022.
33. Dai, K. 2012. Optimization of water and nitrogen use efficiencies in winter wheat and summer maize cropping system in North China Plain. PhD Thesis, Chinese Academy of Agricultural Sciences, Beijing, China.
34. Deng, H. 2006. Study on physiological and ecological characteristics of rice under different irrigation methods. Dissertation of Master Degree, Huazhong Agricultural University, Wuhan, China.
35. Deng, X.Q., Fan, G.G., Zhou, S.L. 2011. Effect of different nitrogen and potassium fertilizer operation on economic characters and yield of potatoes. Soil and Fertilizer Sciences 2: 48–51. (in Chinese with English abstract)
36. Ding, W.X., Cai, Y., Cai, Z.C., Yagi, K., Zheng, X.H., 2007. Nitrous oxide emissions from an intensively cultivated maize–wheat rotation soil in the North China Plain. Science of the Total Environment 373: 501–511.
37. Dong, D.F., Chen, G.X., Ma, L., Liu, F., Yang, Y.J., Ma, W.Q., Wang, P.L. 2013. Research on water-saving irrigation mode for potato. Shandong Agricultural Sciences 45(10): 69–71. (in Chinese with English abstract)
38. Dong, H. 2011. Effects of irrigation and planting patterns on water consumption characteristics and grain yield formation of wheat-maize cropping system. PhD Thesis, Shandong Agricultural

University, Tai'an, China.
39. Dong, L.L., Li, X.P., Jiang, X., Liu, X.C. 2014. Assessment of energy consumption and carbon footprint of rice production using life cycle assessment method: case study in Chongming Island, Shanghai, China. Journal of Agro-Environment Science 33(6):1254–1260. (in Chinese with English abstract)
40. Dou, X.S., Chen, G.R., Gao, S.M., Guo, T.W., Zhang, G.H. 2009. Effect of application of potassium and different stage of water supplement on potato yield in semi-arid area. Research of Agricultural Modernization 30(6): 744–747. (in Chinese with English abstract)
41. Du, J. 2016. Water deficit regulation of oasis potato under mulched drip irrigation and optimizing of the irrigation schedules. Dissertation of Master Degree, Gansu Agricultural University, Lanzhou, China.
42. Duan, P.Z.,Guo, Z., Liu, L., Lin, J.Y. 2014. Current situation, limit factors and development countermeasures of potato production in Hengshui. Agricultural Development and Equipment 11: 23. (in Chinese)
43. Duan, W.X. 2013. Effects of tillage and nitrogen fertilizer management on water consumption characteristics and yield formation in dryland farming Systems of wheat. PhD Thesis, Shandong Agricultural University, Tai'an, China. (in Chinese)
44. Duan, Y., Sun ,G.Q., Zhang, J., Hou, J.W., Li, Y.F., Sha, N., Yun, L.N. 2016. Effect of CRU on tuber yield and N use efficiency of potato. Journal of Northern Agriculture 44(5): 25–30. (in Chinese with English abstract)
45. Fan, S.J., Wang, D., Zhang, J.L., Bai, J.P., Song, J.X., Ma, Z.J. 2012. Effect of tillage strategies on the topsoil water content and the yield of potato. Acta Prataculturae Sinica 21(2): 271–279. (in Chinese with English abstract)
46. Fang, Q.X. 2003. Effects of irrigation on rules of field water consumption and WUE of winter wheat. Dissertation of Master Degree, Shandong Agricultural University, Tai'an, China.
47. Feng, D., Liu, X.F., Kang, Y.Y., Jiang, S.F., Wang, S.Q., Liu, S.P. 2015. Soil water and thermal effects of different mulching and planting methods and their influences on yield in dryland potato production. Water-saving Irrigation 8: 42–44. (in Chinese with English abstract)
48. Feng, K., Ma, Y.M., Jia, L.L., Zhang, J.S., Erken, D., Wang, Z.X., Shi, S.B. 2016. Effects of two irrigation on the agronomic traits and the yield of winter wheat. Xinjiang Agricultural Sciences 53(11): 1999–2007. (in Chinese with English abstract)
49. Feng, R.Y., Yang, W.D., Wang, H.J., Nan, J.F., Zhang, Z.J. 2012. Effects of straw amendment fertilizers on water use efficiency, yield and quality of potato. Transactions of the CSAE 28(2): 100–105. (in Chinese with English abstract)
50. Feng, Y.Q. 2014a. Studies on the rotation and cultivation systems and nitrogen nutrition of potato in winter fallow fields in South China. Dissertation of Master Degree, Hunan Agricultural University, Changsha, China.
51. Feng, Z.Q. 2014b. Analysis of the economic benefit of the early winter potato in mango. Bulletin of Agricultural Science and Technology 2: 24–26. (in Chinese)
52. Feng, Z.W., Kang, Y.W., Wan, S.Q., Liu, S.P. 2016. Effect of cultivation pattern on potato growth and the water and fertilizer efficiency under drip fertigation. Water Saving Irrigation 8: 23–26. (in Chinese with English abstract)
53. Fu, Q. 2000. Research on assembling the saving water technique and synthetically optimum of well irrigation for rice in the course of field produce of Sanjiang Plain. PhD Thesis, Northeast Agricultural University, Harbin, China.
54. Fu, Z.Q., Long, P., Liu, Y.Y., Zhong, J., Long, W.F. 2015. Effects of water and nitrogenous fertilizer coupling on CH_4 and N_2O emission from double-season rice paddy field. Environmental Science 36(5): 3365–3372. (in Chinese with English abstract)
55. Gao, B., Ju, X.T., Meng, Q.F., Cui, Z.L., Christie, P., Chen, X.P., Zhang, F.S. 2015a. The impact of alternative cropping systems on global warming potential, grain yield and groundwater use. Agriculture, Ecosystems and Environment 203: 46–54.
56. Gao, B., Huang, Y.F., Huang, W., Shi, Y.L., Bai, X.M., Cui, S.H., 2018. Driving forces and impacts of food system nitrogen flows in China, 1990 to 2012. Science of the Total Environment 610–611, 430–

441.
57. Gao, L. 2016. Effect of different farming measures on greenhouse gases emission in arid areas farmland. Dissertation of Master Degree, Anhui Agricultural University, Hefei, China.
58. Gao, Y.M., Sun,M., Gao, Z.Q., Cui, K., Zhao, H.M., Yang, Z.P., Hao, X.Y. 2015b. Effects of mulching on grain yield and water use efficiency of dryland wheat in different rainfall years. Scientia Agricultura Sinica48(18):3589–3599. (in Chinese with English abstract)
59. Gao, S.M., Zhang, X.C., Wang, Y.H. 2010. Influence of different mulching and furrow-ridge planting methods on soil moisture and yield of potato on dryland. Journal of Soil and Water Conservation 24(1): 249–256. (in Chinese with English abstract)
60. Gao, X. 2014. Effect of controlled released urea and potassium fertilizers on growth of potato and soil fertility. Dissertation of Master Degree, Shandong Agricultural University, Tai'an, China.
61. Gao, Z.L. 2004. N_2O flux and CH_4 uptake of soil in winter wheat and summer maize rotation system. PhD Thesis, China Agricultural University, Beijing, China.
62. Ge, Q. 2015. Study on the potato and corn cultivation model of integrated and efficient in Hanzhong hilly area. Dissertation of Master Degree, Northwest Agricultural and Forest University, Yangling, China.
63. Gong, C.W., Feng, S.J., Zhao, X.N., Ma, Z.M., Fan, A.P., Yang, J.L. 2013. Effects of different potassium fertilizers on yield and quality of potato in central Gansu Province. Agricultural Research in the Arid Areas 31(3): 112–117. (in Chinese with English abstract)
64. Gu, L.L., Sun, L., Shi, Y., Liu, X.M., Liu, Y.Y. 2013. Effects of nitrogen application time on fry matter accumulation and translocation and tuber yield of potato. Soils 45(4): 610–615. (in Chinese with English abstract)
65. Guan, D.H. 2014. Effect of different water conditions and tillage practices on root growth and water use of winter wheat and summer maize and its chemical regulation. PhD Thesis, China Agricultural University, Beijing, China.
66. Guo, X.P., Jiang, X.H., Yuan, J. 2009. The preliminary research on water consumption, quota of irrigation and drainage and water production efficiency under water-catching and controllable irrigation technology of rice. Journal of Irrigation Drainage 28(1): 65–67. (in Chinese with English abstract)
67. Guo, Y.L., Luo, L.G., Chen, G.X., Kou, Y.P., Xu, H. 2013. Mitigating nitrous oxide emissions from a maize-cropping black soil in northeast China by a combination of reducing chemical N fertilizer application and applying manure in autumn. Soil Science and Plant Nutrition 59(3): 392–402.
68. Guo, Y.M., Guo, X.P., Fan, J.J., Zhang, X.L. 2010. Grain yield and water production efficiency ofrain-water storage and controllable irrigation(RSCI) model of rice. Journal of Irrigation and Drainage 29(3): 61–63. (in Chinese with English abstract)
69. Han, F.X., Chang, L., Chai, S.X., Yang, C.G., Cheng, H.B., Yang, D.L., Li, H., Li, B.W., Li, S.L., Song, Y.L., Lan, X.M. 2016. Effect of straw strip covering on ridges on soil water content and potatoyield under rain-fed semiarid conditions. Chinese Journal of Eco-Agriculture 24(7): 874–882. (in Chinese with English abstract)
70. Hao, S. 2014. A comparative study centering on the influence of organizational degree on the economic benefit of potato production. Dissertation of Master Degree, Inner Mongolia Agricultural University, Inner Mongolia, China.
71. Hao, S.R., Cui, P.C., Zhang, Z.Y., Jin, Y.J., Yang, H.W. 2015. Rice water-saving irrigation quota and suitable irrigation mode in Nanjing. Water Resources Protection 31(6): 150–153. (in Chinese with English abstract)
72. He, J.Y., Tian, J.C., Ma, B., Shen, H. 2015. Water production function of paddy rice in aerobic soil with drip irrigation under plastic film. Acta Agriculturae Boreall-occidentalis Sinica 24(1): 41–48. (in Chinese with English abstract)
73. Hou, X.Q., Li, R., He, W.S., Ma, K., Dai, X.H. 2015. Effect of super absorbent dosages on physical-chemical proporties od dryland soil and potato growth. Journal of Soil and Water Conservation 29(5): 325–330. (in Chinese with English abstract)
74. Hou, X.Q., Tang, J., Yu, L.L., Zhao, F.P., Wang, Q.W., Hu, E.J., Wei, K.R. 2016. Effect of autumn mulching tillage on growth and water use efficiency of potato. Journal of drainage and irrigation

machinery engineering (JDIME) 34(2) : 165–172. (in Chinese with English abstract)
75. Hou, X.Y., Wang, F.X., Kamg, S.Z., Feng, S.Y., Han, J.J. 2008. Effects of uncovering plastic film mulch on drip-irrigated potato in arid Minqin oasis, Northwest China. Agricultural Research in the Arid Areas 26(4): 87–93. (in Chinese with English abstract)
76. Hu, J.Q., Liu, H.G. 2012. Study and discussion on cold rice water saving irrigation control technology. North Rice 42(6): 47–50. (in Chinese)
77. Hu, X.K., Su, F., Ju, X.T., Gao, B., Oenema, O., Christie, P., Huang, B.X., Jiang, R.F., Zhang, F.S. 2013. Greenhouse gas emissions from a wheat-maize double cropping system with different nitrogen fertilization regimes. Environmental Pollution 176: 198–207.
78. Hu, T., Tong , Y.A., Gao, P.C., Gao, B., Ju, X.T. 2014. N_2O emission characteristics and mitigation methods in South Loess Plateau under rain-fed winter wheat conditions. Chinese Journal of Eco-Agriculture 22(9): 1038─1046. (in Chinese with English abstract)
79. Huang, C.B., Zhong, Z.Z., Xie, L.H. 2012. Preliminary report on the fertilizer efficiency experiment "3414"of potato in Xiapu County, Fujian Province. Subtropical Agriculture Research 8(1): 13–16. (in Chinese with English abstract)
80. Huang, C.J., Zhao, S.Y., Wang, L.C., Wang, J.C., Zhao, Y., Cai, Y.M., Teng, Y., Yang, G.C. 2013a. Effect of potato/maize intercropping on photosynthetic characteristics and yield in two potato varieties. Acta Agronomica Sinica 39(2): 330–342. (in Chinese with English abstract)
81. Huang, J.C., Peng, Z.P., Yu, J.H., Wu, X.N., Yang, L.X., Lin, Z.J. 2014. Effect of potassium application rate on yield, quality and potassium efficiency of winter potato. China Potato Journal 30(19): 167–171 (in Chinese with English abstract)
82. Huang, J.X., Sui, P., Li, Y.Y., Gao, W.S., Chen, Y.Q. 2011b. Assessment of environmental impact on different conservation tillage patterns in double-crop rice area in Hunan province. China Population, Resources and Environment 21(11):124–130. (in Chinese with English abstract)
83. Huang, M. 2014. The research of non-point source pollution of paddy by different combination models of water and fertilizers. Dissertation of Master Degree, Jiangxi Agricultural University, Nancang, China.
84. Huang, T., Gao, B., Christie, P., Ju, X.T. 2013b. Net global warming potential and greenhouse gas intensity in a double cropping cereal rotation as affected by nitrogen and straw management. Biogeosciences 10: 1–15.
85. Huang, X.Y., Xu, Y.C., Shen, Q.R., Zhou, C.L., Yin, J.L., Dittert, K. 2003. Water use efficiency of rice crop cultivated under waterlogged and aerobic soil mulched with different materials. Journal of Soil and Water Conservation 17(3): 140–143. (in Chinese with English abstract)
86. Huang, X.Y. 2004. Growth and water use efficiency of rice cultivated under non-flooded soil condition mulched with different materials. Dissertation of Master Degree, Nanjing Agricultural University, Nanjing, China.
87. Huang, Z.D., Qi, X.B., Fan, X.Y., Hu, C., Zhu, D.H., Li, P., Qiao, D.M. 2010. Effect of alternate partial root-zone subsurface drip irrigation on potato yield and water use efficiency. Chinese Journal of Applied Ecology 21(1): 79–83. (in Chinese with English abstract)
88. Huang, Z.G., Wang, X.L., Xiao, Y. Yang, F., Wang, C.X. 2015. Effect of climate change on rice irrigation water requirement in Songnen Plain, Northeast China. Chinese Journal of Applied Ecology 26(1): 260–268. (in Chinese with English abstract)
89. Huo, X.L., Meng, Q.Y., Hao, B.P., Hua, X.Z., Zhao, X.F. 2016. Analysis of the status quo and problems of potato nutrient management in Shanxi. Journal of Shanxi Agricultural Sciences 44(7): 1033–1036. (in Chinese with English abstract)
90. IPCC. 2001. Good practice guidance and uncertainty management in national greenhouse gas inventories. Published. Japan: IGES. 2001.
91. Ji, F. 2008a. Water requirement rules and water use efficiency of rice under different water treatments. Dissertation of Master Degree, Northeast Agriculture University, Haerbin, China.
92. Ji, S.L. 2008b. Comparative analysis of production cost and benefit of the three crops in southern mountain area of Ningxia. Modern Agricultural Science and Technology 16: 224–225. (in Chinese)
93. Ji,Y. 2008b. Study on optimized fertilization and potassium effect on detoxification potato in high altitude region. Dissertation of Master Degree, Guizhou University, Guizhou, China.

94. Jia, L. 2012. Study on high-efficiency water and fertilizer management of winter wheat under limited irrigation conditions in Weibei plateau. Dissertation of Master Degree, Northwest Agricultural &Forest University, Shaanxi, China.
95. Jia, L., Chen, X., Zhang, F., Buerkert, A., Röemheld, V. 2007. Optimum nitrogen fertilization of winter wheat based on color digital camera images, Communications in Soil Science and Plant Analysis 38(11): 1385–1394.
96. Jia, Z.Y., Zhu, Q.W., Yang, F. 2012. Plastic film mulching cultivation: technology for resource saving water of potato in Mid-Southern areas of Ningxia. Ningxia Journal of Agricultural and Forest Science and Technology 53(12):28, 49. (in Chinese with English abstract)
97. Jiang, C.S. 2005. Study on emission of major greenhouse gases from agro-ecosystems in a central Sichuan hilly area of Southwest China. PhD Thesis, Chinese Academy of Sciences, Beijing, China.
98. Jiang, D.Y., Yu, Z.W. 2007. Effect of water and nitrogen cooperation on grain yield and water use Efficiency in winter wheat. Hubei Agricultural Sciences 46(5): 699–700. (in Chinese with English abstract)
99. Jiang, X.D., Li, Z.J., Hou, L.T., Wang, Y., Wang, X., Yan, H. 2005. Impacts of minimum tillage and no-tillage systems on soil NO_3^--N content and water use efficiency of winter wheat/summer corn cultivation. Transactions of the CSAE 21(7): 20–24. (in Chinese with English abstract)
100. Jin, X.X., Zhang, X.Y., Chen, S.Y., Sun, H.Y., Wang, Y.M., Shao, L.W., Gao, L.N. 2009. Effect of different irrigation frequency and amount on nitrogen uptake, translocation of winter wheat. Acta Agriculturae Boreali-Sinica 24(4): 112–118. (in Chinese with English abstract)
101. Jing, T., Fan, M.S., Zhou, D.B., Qin, Y.L., Shi, X.H. 2012. Effects of nitrogen fertilization on potato tuber yield, N uptake and soilNO_3^--N accumulation under plastic mulching with drip irrigation. Plant Nutrition and Fertilizer Science18(3): 654–661. (in Chinese with English abstract)
102. Jing, Z.Z., Li, Z.G., Wu. G.Q., Li, Y.Z. 2009. The main diseases of the potato in Ningnan mountainous area and the protection technology of non-public nuisance. Chinese hi-tech enterprises 12: 15–16. (in Chinese)
103. Ju, X.T., Lu, X., Gao, Z.L., Chen, X.P., Su, F., Kogge, M., Römheld, V., Christie, P., F.S. Zhang. 2011. Processes and factors controlling N_2O production in an intensively managed low carbon calcareous soil under sub-humid monsoon conditions. Environmental Pollution 159: 1007–1016.
104. Ke, C.Y. 2010. Effect of different water treatment on rice growth, yield and quality. Dissertation of Master Degree, Huazhong Agricultural University, Wuhan, China.
105. Kong, L., Zhu, L.Z. 2016. Study on carbon emissions from potato production—Empirical analysis based on agricultural inputs and LMDI models. 7: 111–121. (in Chinese)
106. Kong, L.Y., Peng, Q.S., Xiong, Y., Zhou, F.C. 2004. Effect of balanced fertilization on the yield and quality of potato. Soil and fertilizers 3: 17–19. (in Chinese with English abstract)
107. Kong, X.W., Liu, Y.L., Xiong, Z.Q., Ma, Y.C., Zhang, X.L., Qin, J.Q., Tang, Q.Y. 2013. CH_4 and N_2O emissions from double rice field under different intensified cultivation patterns in Hunan province. Acta Scientiae Circumstantiae 33(9): 2612–2628. (in Chinese with English abstract)
108. Lan, X.M., Huang, C.X., Li, B.W., Li, S.L., Song, Y.L., Chai, Y.W., Cheng, H.B., Chang, L., Chai, S.X. 2016. Effect of mulching materials on soil temperature and yield of winter wheat in Northwest China. Journal of Triciteae Crops 36(8): 1084–1092. (in Chinese with English abstract)
109. Lei, W.S., Wang, Y.P. 2013. Effect of different nitrogen management strategies on nitrogen uptake, utilization and yield of spring potato in hilly regions. Agricultural Sciences of Guangdong 21: 61–65. (in Chinese with English abstract)
110. Li, B., An, J.W., He, Z.G., Lou, C.R. 2014a. The effect of controlled release fertilizer on the production of potato and variations of soil available nutrient contents. Liaoning Agricultural Sciences 6: 30–33. (in Chinese with English abstract)
111. Li, C.F., Zhang, Z.S., Guo, L.J., Cai, M.L., Cao, C.G. 2013a. Emissions of CH_4 and CO_2 from double rice cropping systems under varying tillage and seeding methods. Atmospheric Environment 80: 438–444
112. Li, C.H., Zeng, K., Wei, S.Q., Xu, S.H., Jiang, L.G. 2011a. Water use of rice under different soil tillage and irrigation patterns. Crops 4: 81–84. (in Chinese with English abstract)
113. Li, F., Miao, Y.X., Zhang, F.S., Cui, Z.L., Li, R., Zhang, H.Y, Schroder, J., Raun, W.R., Jia, L.L. 2009a.

In-season optical sensing improves nitrogen-use efficiency for winter wheat. Soil Science Society of American Journal 73: 1–9.

114. Li, F.X., Yuan, H.Y., Yan, X.H., Li, M.T. 2005. Study on climate ecology for rice plant height under different irrigation treatments in Ningxia irrigation area. Arid Meteorology 23(3): 62–64. (in Chinese with English abstract)

115. Li, G.P., Zhang, S.L., Yuan, J.C., Sun, L., Liu, L., Zhou, H. 2009b. Optimizing methods analysis of nitrogen application strategies on spring potato of high yield, good quality and high efficiency in Ya'an region. Southwest China Journal of Agricultural Science 22(3): 707–711. (in Chinese with English abstract)

116. Li, H.L., Wang, D.S., Ji, X.Q., Wang, Y. 2014. Yield analysis of sowing small whole seed potatoes under different phosphorus fertilizer levels. China Potato Journal 28(5): 292–294. (in Chinese with English abstract)

117. Li, J.J., Pan, G.X., Li, L.Q., Zhang, X.H. 2009c. Estimation of net carbon balance and benefits of rice-rice cropping farm of a red earth paddy under long term fertilization experiment from Jiangxi, China. Journal of Agro-Environment Science 28(12): 2520–2525. (in Chinese with English abstract)

118. Li, K. 2006. Study on the soil microbial biomass and greenhouse gas emission from paddy-soil system. Dissertation of Master Degree, Sichuan Agricultural University, Sichuan, China.

119. Li, P., Qi, X.B., Fan, X.Y., Wu, H.Q., Qiao, D.M., Fan, T., Zhao, Z.J., Zhuang, Z.D., Zhu, D.H. 2009a. Effect of alternate partial root-zone irrigation on nitrogen and water use efficiency of potato. Transactions of the CSAE25 (6): 92–95. (in Chinese with English abstract)

120. Li, Q., Liu, J.H., Zhang, L., Chen, Q., Yu,J., Achary, S.N. 2013b. Using water-retaining agent and mulch to improve growth and yield of potato under dry farming. Transactions of the CSAE 29(7):83–90. (in Chinese with English abstract)

121. Li, R., Hou, X.Q. 2015. Effects of different ground surface mulch under subsoiling on potato yield and water use efficiency. Transactions of the CSAE 31(20): 115–123. (in Chinese with English abstract)

122. Li, S. 2016a. Effect of different irrigation modes on yields and water use efficiency in potato system. Gansu Agriculture 12: 17–18 (in Chinese)

123. Li, S.S., Li, Q.M., Zhang, H.M., Han, Y.S., Li, Y. 2007. Quality and efficient soil testing technology research and application of applying fertilizer on potato. Food and Nutrition in China 8: 17–19.

124. Li, X.M. 2014. Study on greenhouse gas emissions and its influencing factors from the maize-wheat rotation field. Dissertation of Master Degree, Beijing Forestry University, Beijing, China.

125. Li, Y., Lv, D.Q., Hu, S.L., Gu, H.B., Qu, L., Wang, S.P., Liu, S.W., Chen, J.X., Yang, H.C. 2013. Influence of various NPK combinations on agronomic trait, yield and dry matter content in potato. China Potato 27(3): 1148–1152. (in Chinese with English abstract)

126. Li, Y., Yang, X.G., Ye, Q., Huang, W.H. 2011b. Variation characteristics of rice water requirement in middle and lower reaches of Yangtze River during 1961-2007. Transactions of the CSAE 27(9): 175–183. (in Chinese with English abstract)

127. Li, Y.J. 2012. Studies on effects and mechanisms of water conditions and nitrogen fertilizer levels on nitrogen use efficiency in a rice cropping system. PhD Thesis, Zhejiang University, Hangzhou, China.

128. Li, Y.J., Wu, J.Z., Huang, M., Yao, Y.Q., Zhang, C.J., Cai, D.X., Jin, K. 2006a. Effects of different tillage systems on photosynthesis characteristics of flag leaf and water use efficiency in winter wheat. Transactions of the CSAE 22(12): 44–48. (in Chinese with English abstract)

129. Li, Y.P., Wang, T.Y., Liu, S.X., Liu, X.P., Jia, Z.K., Ren, S.C. 2006b. Effects of compound fertilizers specified for potato and water-fertilizer production efficiency. Chinese Agricultural Science Bulletin 22(11): 194–197. (in Chinese with English abstract)

130. Li, Y.S., Bai, J.M., Xu, S.K., Pu, H.M., Wang, Y.H., Ji, S.T., Xie, F.S., Sui, Q.J. 2015. Effects of different irrigation amount on winter potato growth and water use efficiency by drip irrigation under film. Agricultural Research in the Arid Areas 33(6): 8–13. (in Chinese with English abstract)

131. Li, Z.H. 2016b. Effect of energy use on production and income: evidence from potato production area of northern China. PhD Thesis, Chinese Academy of Agricultural Sciences, Beijing, China.

132. Liang, C.Y. 2009. The economic benefit analysis and the high yield culture technique of virus-free potato. Journal of Agriculture 10: 27–31. (in Chinese)

133. Liang, G., Liang, L.Z., Dong, X.Y., Shen, R.F. 2016. Effects of controlled-release fertilizer on wheat-

maize rotation system in fluvo-aquic soil in North China. Soils48(1): 53–58. (in Chinese with English abstract)
134. Liang, J.X., Guo, X.N., Zhang, G.H., Zhou, T. 2015. Effect of different N, P_2O_5, K_2O rates on yield and water, fertilizer using efficiency of potato in dry land of Ningnan. Soil and Fertilizer Sciences6: 76–81. (in Chinese with English abstract)
135. Liang, L., Chen, Y.Q., Gao, W.S., Sui, P., Chen, D.D., Zhang, W. 2009. Life cycle environmental impact assessment in winter wheat-summer maize system in North China Plain. Journal of Agro-Environment Science 28(8): 1773–1776. (in Chinese with English abstract)
136. Liang, M.Z., Tan, Z.C., Chen, L.B., Zhou, G.Q., Lei, B.H., Jiang, Z.Y. 2000. Influence of drought stress on water use efficiency of paddy rice. Life Science Research 4(4): 351–355. (in Chinese with English abstract)
137. Liang, X., Zhang, S., Meng, M.L., Yue, H.L., Liu, W.L. 2013. Effects of N additional fertilization on nitrogen absorption and accumulation and use efficiency of potato under mulched drip irrigation. 27(1): 42–47. (in Chinese with English abstract)
138. Liao, J.L. 2009. Effect of water and fertilizers managing on potato growth and WUE and soil fertility in dry land. Dissertation of Master Degree, Northwest Agriculture & Forestry University, Yangling, China.
139. Lin, X.Q., Zhou, W.J., Zhu, D.F., Zhang, Y.P. 2004. Effect of water management on photosynthetic rate and water use efficiency of leaves in paddy rice. Chinese Journal of Rice Science 18(4): 333–338. (in Chinese with English abstract)
140. Lin, Y.C., Bu, H.Z., Hu, Y.G., Zeng, Z.H., Xiao, X.P., Tang, H.M., Yang, G.L. 2012. Effects of different cover types on soil water, temperature and potato yield in double rice cropping area. Research of Agricultural Modernization 33(2): 216–220. (in Chinese with English abstract)
141. Liu, C.Y., Wang, K., Meng, S.X., Zheng, X.H., Zhou, Z.X., Chen, D.L., Yang, Z.P. 2011a. Effects of irrigation, fertilization and crop straw management on nitrous oxide and nitric oxide emissions from a wheat–maize rotation field in northern China. Agriculture, Ecosystems and Environment 140: 226–233.
142. Liu, C.Y., Yao, Z.S., Wang, K., Zheng, X.H. 2014. Three-year measurements of nitrous oxide emissions from cotton and wheat-maize rotational cropping systems. Atmospheric Environment 96: 201-208.
143. Liu, D.X. 2013a. Optimal fertilization reduced greenhouse gas emissions of wheat-maize cropping system. Dissertation of Master Degree, Shandong Agricultural University, Tai'an, China.
144. Liu, F., Zhuge, Y.P., Chen, Z.M., Wang, H., Zhu, L. 2011b. Effects of controlled-release fertilizer on potato yield, nitrogen use efficiency and economic benefit. Chinese Agricultural Science Bulletin27(12):215–219. (in Chinese with English abstract)
145. Liu, G.H., Su, Y., Feng, Z.W. 2009a. Analysis on economic benefit of winterness potato planted in rice paddy under no-tillage with rice straw mulch cultivation. Guizhou Agricultural Sciences 37(5): 64–65. (in Chinese with English abstract)
146. Liu, G.M., Yang, J.S., Jiang, Y., Zhang, X.Y. 2005. Study of water-demand regulations and water use efficiencies of rice under condition of different water-saving irrigation systems. Journal of Irrigation and Drainage 24(6): 49–52. (in Chinese with English abstract)
147. Liu, H.T. 2014. Evaluating soil profile properties and water nitrogen use efficiencies of high yield region in the North China Plain. PhD Thesis, China Agricultural University, Beijing, China.
148. Liu, L.G., Wu, X., Wang, L.H., Yang, X.W. 2016. Rice evapotranspiration and growth characteristics under different water saving irrigation models in northern Hubei province. Journal of Irrigation Drainage 35(3): 32–36. (in Chinese with English abstract)
149. Liu, M. 2015. The effects of nitrogen application rate and method on N_2O emission in winter wheat-summer maize rotation. PhD Thesis, China Agricultural University, Beijing, China.
150. Liu, M., Yang, S.H., Xun, J.Z., Peng, S.Z. 2014. Effect of controlled released fertilizer on rice yield, water and nitrogen use efficiency under water-saving irrigation. Water Saving irrigation 5: 7–10. (in Chinese with English abstract)
151. Liu, R.L., Li, Y.H., Wang, F., Chen, Z.J., Zhao, T.C., Chen, C. 2009b. Effect of different style potassium fertilizer on potato nutrient elements accumulation and yield. Acta Agriculturae Boreali-occidentalis

Sinica 18(1):143–146. (in Chinese with English abstract)
152. Liu, R.M., Fan, M.P., F, Y.Z., Zhou, J., Zheng, Y., Tang, L. 2014. Relationship of fertilization rate and fertilizer partial factor productivity of potato production in Yunnan province. Acta Pedologica Sinica 51(4): 753–760. (in Chinese with English abstract)
153. Liu, X.J. 2011. Effect of different water and nitrogen treatments on rice growth and yield formation. Dissertation of Master Degree, Huazhong Agricultural University, Wuhan, China.
154. Liu, Y.F. 2012. Rice physiology and yield and microbial ecology in paddy soil under different irrigation methods and fertilization. PhD Thesis, Guangxi University, Nanning, China.
155. Liu, Z., Qin, S.J., Wang, D., Zhang., J.L. 2010b. Effects of limited supplementary irrigation using catchment rainfall on accumulation and distribution of dry matter and yield of potato in Longzhong semiarid area. Agricultural Research in the Arid Areas 28(4): 46–49. (in Chinese with English abstract)
156. Liu, Z.D., Xiao, J.F., Yu, X.Q. 2010a. Effect of different water treatment on morphological index, water consumption and quality of potatoes. China's Rural Water Conservancy and Hydropower 8: 1–3. (in Chinese with English abstract)
157. Liu, Z.X. 2013. Effects of tillage practices on greenhouse gases emissions from a Purple Soil under rice-rape rotation system. Dissertation for Master Degree, Southwest University, Chongqing, China.
158. Long, P. 2014. Effects of organic wastes incorporation on soil organic carbon and net carbon balance in wheat-maize farming system. PhD Thesis, China Agricultural University, Beijing, China.
159. Lu, Y., Huang, H., Zheng, H.B., Yao, L., He, H., Liu, J.X., Li, J.Y. 2015. Evaluation of the carbon sequestration capacity and carbon footprints in the rice production systems-A case of Hunan province. Crop Research 29(3): 240–243 (in Chinese with English abstract)
160. Luo, Y.F., Peng, S.Z., Wang, W.G., Jiao, X.Y., Sun, Y., Han, B. 2009. Impacts of climate change on irrigation water requirements of rice: a case study of Gaoyou Irrigation District. Engineering Journal of Wuhan University 42(5): 609–613. (in Chinese with English abstract)
161. Lv, H.F., Wang, X.J., Chen, Y., Lu, X.Y., Mao, G.Q., Shen, Y.S. 2010. Effects of nitrogen, phosphorus and potassium application by stages on yield and quality of potato. Chinese Agricultural Science Bulletin26(24):197–200. (in Chinese with English abstract)
162. Lv, L., Feng, C.P., Cui, Y.L. 2011. Comparison of water-using efficiency for paddy rice under different water saving irrigation method. Water Saving Irrigation 3: 15–17. (in Chinese with English abstract)
163. Ma, F.Y., Zhang, Y.C., Tian, F., Zhang, F.J. 2011. Effect of different fertilizer amount and density on potato yield in alpine-cold region. Journal of Changjiang Vegetables 2: 55–58. (in Chinese with English abstract)
164. Ma, H.L., Guo, Z.P. 2007. Study on fertilization measures for high-yielding potato. China Potato Journal 21(1): 26–28. (in Chinese with English abstract)
165. Ma, L., Ma, D, Zhang, F. 2011. Application effects of various irrigation methods on potato production. China Potato 25(2): 89–91. (in Chinese with English abstract)
166. Ma, S.Y., Yu, Z.W., Zhang, Y.L., Zhao, J.Y., Shi, Y., Wang, D. 2014. Effect of field border width for irrigation on water consumption characteristics, yield and water use efficiency of wheat. Scientia Agricultura Sinica 47(8):1531–1540. (in Chinese with English abstract)
167. Ma, X.H., Wang, D., Yu, Z.W., Wang, X.Z., Xu, Z.Z. 2010. Effect of irrigation regimes on water consumption characteristics and nitrogen distribution in wheat at different nitrogen applications. Acta Ecologica Sinica 30(8):1955–1965. (in Chinese with English abstract)
168. Ma, Y.L., Ji, Y.Z., Li, X., Zhang, L., Ju, X.T., Zhang, L.J. 2012. Effects of N fertilization rates on the NH_3 volatilization and N_2O emissions from the wheat-maize rotation system in North China Plain. Ecology and Environmental Sciences 21(2): 225–230. (in Chinese with English abstract)
169. Mai, Z.Z., She, P., Mai, J., Wang, Y., Zhou, H.L., Mi, Z.M., Lu, J.W., Jin, X.P. 2014. Effects of mulching timing, pattern and color of plastic film on soil moisture and water use efficiency of potato. Agricultural Research in the Arid Areas 32(1): 1–10. (in Chinese with English abstract)
170. Meng, Q.F., Sun, Q.P., Chen, X.P., Cui, Z.L., Yue, S.C., Zhang, F.S., Römheld, V. 2012. Alternative cropping systems for sustainable water and nitrogen use in the North China Plain. Agriculture Ecosystems and Environment 146: 93–102.
171. MOA (Ministry of Agriculture of China). 2015 China Agriculture Yearbook. China Agriculture Press,

Beijing, China.
172. Mu, J.X., Cao, X.M., Liu, S.C. 2016. Effects of combined application of water-retaining agent and nitrogen fertilizer on growth and water and fertilizer utilization of potato. Journal of Henan Agricultural Sciences 45(9): 35–40. (in Chinese with English abstract)
173. Nie, Z.J., Chen, Y.Q., Zhang, J.S., Shi, J.T., Li, C., Gao, W.S., Sui, P. 2013. Effects of drip irrigation patterns on wheat yield and water use efficiency in Heilonggang region. Acta Agronomica Sinica 39(9): 1687–1692. (in Chinese with English abstract)
174. Ning, D.F. 2010. Study on winter wheat cultivation techniques with higher water and nitrogen utilization efficiency in Huang-Huai-Hai area of China. Dissertation of Master Degree, Chinese Academy of Agricultural Sciences, Beijing, China.
175. NHFPC (National Health and Family Planning Commission). 2015. Chinese residents dietary nutrition and chronic disease status reports. People's Medical Publishing House, Beijing, China, 2015.
176. Pan, X.L. 2012. Effect of irrigation and fertilization on water and nitrogen use of intensive wheat-maize double cropping system. Dissertation of Master Degree, Chinese Academy of Agricultural Sciences, Beijing, China.
177. Pei, S.W. 2012. The effect of fertilization on greenhouse gas and nitrogen gas emissions from the field in North China Plain. Dissertation of Master Degree, Beijing Forestry University, Beijing, China.
178. Peng, C.R. 2012. Study on the techniques of “three high one ensure” cultivation and its mechanism of high yielding and quality in rice. PhD Thesis, Jiangxi Agricultural University, Nancang, China.
179. Peng, H., Ji, X.H., Wu, J.M., Zhu, J., Huang, J. 2015b. CH_4 and N_2O emission reduction under different cropping systems in double-cropping paddy fields. Ecology and Environmental Sciences 24(2): 190–195. (in Chinese with English abstract)
180. Peng, Y., Sun, Y.J., Jiang, M.J., Xu, H., Qin, J., Yang, Z.Y., Ma, J. 2014. Effects of water management and slow/controlled release nitrogen fertilizer on biomass and nitrogen accumulation, translocation, and distribution in rice. Acta Agronomica Sinica 40(5): 859–870. (in Chinese with English abstract)
181. Peng, Z.P., Liu, Y.L., Li, Y.C., Wang, Y.Q., Shu, X.X., Wei, S.S., Men, M.X., Liu, H.L. 2015a. Effect of constant nitrogen regulation on the nitrogen utilization and apparent loss in the rotation system of wheat and maize. Journal of Soil and Water Conservation 29(6): 74–79. (in Chinese with English abstract)
182. Qin, J.H., Chen, Y.J., Zhou, C.Y., Pang, L.P., Meng, M.L. 2013. Effects of drip irrigation frequency under mulch on potato growth, yield and water use efficiency. Chinese Journal of Eco-Agriculture21(7): 824–830. (in Chinese with English abstract)
183. Qin, S.J., Zhang, J.L., Wang, D., Pu, Y.L., Du, Q.Z. 2011. Effects of different film mulch and ridge-furrow cropping patterns on yield formation and water translocation of rain-fed potato. Chinese Journal of Applied Ecology 22(2): 389–394. (in Chinese with English abstract)
184. Qin, S.L. 2013. Water and fertilizer use efficiencies of potato under different irrigation patterns and nitrogen recommendation of under-mulch-drip irrigated potato. PhD Thesis, Inner Mongolia Agricultural University, Inner Mongolia, China.
185. Qin, X., Liu, K., Zhou, L.L., Zhou, S.L., Lu, L.Q., Wang, R.Z. 2012. Characteristics of annual water utilization in winter wheat summer maize rotation system in North China Plain. Scientia Agricultura Sinica 45(19):4014–4024. (in Chinese with English abstract)
186. Qin, X.B. 2011. Mitigation of greenhouse gas intensity from typical double rice field of central China. PhD Thesis, Chinese Academy of Agricultural Sciences, Beijing, China.
187. Qin, X.B., Li, Y.E., Liu, K.Y., Wan, Y.F. 2006. Methane and nitrous oxide emission from paddy field under different fertilization treatments. Transactions of the CSAE 22(7): 143–148. (in Chinese with English abstract)
188. Qin, X.B., Li, Y.E., Wan, Y.F., Liao, Y.L., Fan, M.R., Gao, Q.Z., Liu, S., Ma, X. 2014. Effect of tillage and rice residue return on CH_4 and N_2O emission from double rice field. Transactions of the CSAE 30(11): 216–224. (in Chinese with English abstract)
189. Qiu, X.Q., Huang, L., Gao, Y., Duan, A.S., Gong, W.J., Liang, C.S. 2012. Analysis of difference in water use efficiency of winter wheat cultivars released in different eras. Journal of Irrigation and Drainage 31(2): 25–29. (in Chinese with English abstract)

190. Ren, Y.F., Zhao, P.Y., Zhao, J., lu, Z.Y., Zhang, Y.P. 2013. Effects of different treatments of tuber on the growth and development of potato in dryland. Crops 6: 143–145. (in Chinese with English abstract)
191. Shang, Q.Y., Yang, Q.X., Gao, C.M., Wu, P.P., Liu, J.J., Xu, Y.C., Shen, Q.R., Zou, J.W., Guo, S.W. 2011.Net annual global warming potential and greenhouse gas intensity in Chinese double rice-cropping systems: a 3-year field measurement in long-term fertilizer experiments. Global Change Biology 17: 2196–2210.
192. Shang, Q.Y., Yang, X.X., Cheng, C., Luo, K., Huang, S.S., Shi, Q.H., Pan, X.H., Zeng, Y.J. 2015. Effects of water regimes on yield-scaled global warming potential under double rice-cropping systems with straw returning. China Journal of Rice Science 29(2): 181–190.
193. Shang, W.Y., Li. R.M., Liu, H.S., Zhang, X.Y., Ji, B.X., Chen, W.X., Qiu, G.Y. 2016. Phosphorus application amount effect on coated potato in mid- southern of Chengde, Heibei. Chinese Horticulture Abstracts pp: 2–5.
194. Shao, X.H., Zhang, Z.Y., Zhou, M.Y., Jin, B.B. 2003. Water-saving irrigation regime and nitrogen fertilizer application for rice fields under seepage irrigation. Shuili Xuebao 5: 107–111. (in Chinese with English abstract)
195. Shen, J.J., Wang, J., Pan, X.B., Feng, L.P., Zhao, P.Y., Chen, C. 2012. Effect of sowing date on the growth and yield formation and water use efficiency of potato in agro-pastoral ecotone. Agricultural Research in the Arid Areas 30(2): 137–144. (in Chinese with English abstract)
196. Shi, L.G., Chen, F., Kong, F.L., Fan, S.C. 2011c. The carbon footprint of winter wheat-summer maize cropping pattern on North China Plain. China Population, Resources and Environment 21(9): 93–98. (in Chinese with English abstract)
197. Shi, S.Q., Zhao, Y., He, Z.G., Lou, C.R. 2016. The effect of bio-organic fertilizer with chemical fertilizer on soil nutrient migration and yield of potato. Jiangsu Agricultural Sciences 44(6): 154–157. (in Chinese)
198. Shi, S.W., Li, Y.E., Li, M.D., Wan, Y.F., Gao, Q.Z., Peng, H., Qin, X.B. 2011b. Annua1 CH_4 and N_2O emissions from double rice cropping systems under various fertilizer regimes in Hunan province, China. Chinese Journal of Atmospheric Sciences 35(4): 707–720. (in Chinese with English abstract)
199. Shi, S.W., Li, Y.E., Wan, Y.F., Qin, X.B., Gao, Q.Z. 2011a. Effect of the changes in N and P_2O_5 application on CH_4 and N_2O emissions from double rice systems. Environmental Science 32(7): 1899–1907. (in Chinese with English abstract)
200. Shi, Y.F., Wu, W.L., Meng, F.Q., Zhang, Z.H., Zheng, L., Wang, D.P. 2013a. Integrated management practices significantly affect N_2O emissions and wheat–maize production at field scale in the North China Plain. Nutrient Cycling in Agroecosystems DOI 10.1007/s10705-013-9558-9.
201. Shi, Y.L. 2014. Study on the efficiency and adjustment of reactive nitrogen cascade flow of food chain in China. PhD Thesis, Chinese Academy of Sciences, Beijing, China.
202. Shi, Y.T., Chen, Y.L., Liu, S.H., Hou, Y.Q., Pei, H.D., Wang, H.H., Zhang, Y.P. 2013b. Influence of different mulching models on soil moisture and temperature, and yield of potato in semi-arid land. China Potato 27(1): 19–24. (in Chinese with English abstract)
203. Shu, C.L., Ma, X.Z., Meng, M.L., Fan, X.S., Ren, S.Y., Zhang, Q. 2017. Effect of biochar based fertilizer amendment on soil properties and greenhouse emission. Journal of Inner Mongolia Agricultural University (Natural Science Edition). 38(2): 49–61. (in Chinese with English abstract)
204. Song, N., Wang, F.X., Yang, C.F., Yang, K.J. 2013. Coupling effects of water and nitrogen on yield, quality and water use of potato with drip irrigation under plastic film mulch. Transactions of the CSAE29(13): 98–105. (in Chinese with English abstract)
205. Su, X.J., Wang, P., Liu, S.Y., Gao, X.L., Fan, X. 2010. Effect of fertilization on the nutrient uptake dynamic, yield and quality of potato in Dingxi region. Acta Agriculturae Boreali-occidentalis Sinica 19(1): 86–91. (in Chinese with English abstract)
206. Sun, A.H. 2011. Study on water-fertilizer effect and irrigation methods of rice in Sanjiang Plain. PhD Thesis, Northeast Agricultural University Harbin, Heilongjiang, China.
207. Sun, C.M. 2005. Study on technology system of high yield and high value of spring potato in Taixing region. Dissertation of Master Degree, Nanjing Agricultural University, Nanjing, China.
208. Sun, J.M., Guo, Y.Z., Miao, S.Q., 2015. Improvement of potatoes steamed bread to staple foods

nutrition structure of Chinese resident. Journal of Agricultural Science and Technology 17(6): 64–69.(in Chinese with English abstract)
209. Sun, Y.J. 2010. Effects of water-nitrogen interaction on yield formation and characteristics of nitrogen utilization in rice and its physiological basis. PhD Thesis, Sichuan Agricultural University, Wenjiang, Sichuan, China.
210. Tan, Q.K., Li, H.S., Lin, J., Deng, P.L., Zheng, R.F., Zheng, W.P., Chen, J.Z. 2012. Effect of different fertilization level on the agronomic traits and yield of potato. Chinese Agricultural Science Bulletin 28(33):166–171. (in Chinese with English abstract)
211. Tan, X.L., Lv, J.F., Guo, T.W., Guo, X.S., Zhang, X.C., Zhang, P.L. 2011. Effects of plastic film mulching and fertilization on potato dry matter accumulation and soil water content in dryland. Journal of Irrigation and Drainage 30(2): 104–106. (in Chinese with English abstract)
212. Tang, Q.X., Lin, T., Dong, W.J., Shi, S.B. 2015. Analysis of winter wheat yield formation and water use efficiency of drip irrigation under drought condition. Xinjiang Agricultural Sciences52(3): 429–435. (in Chinese with English abstract)
213. Tang, R.Y., Gao, S.M., Wang, Y.H., Zhang, X.C. 2013. Soil water and thermal effects of different mulching and planting methods and their influences on yield in dryland potato production. Agricultural Research in the Arid Areas 33(1): 1–13. (in Chinese with English abstract)
214. Tang, Y.P., Li, W.J., He, X.M., Tan, G.N., Xie, K.Y. 2012. Effect of different rice-straw mulch and planting patterns on the yield of winter potato in Guangxi. China Potato Journal 3: 147–154. (in Chinese with English abstract)
215. Tian, S.Z., Wang, Y., Ning, T.Y., Zhao, H.X., Wang, B.W., Li, N., Li, Z.J., Chi, S.Y. 2013. Greenhouse gas flux and crop productivity after 10 years of reduced and no tillage in a wheat-maize cropping system. PLoS ONE 8(9): e73450. doi:10.1371/journal.pone.0073450.
216. Tian, Y., Li, F.M., Liu, X.L. 2007. Effects of different ridge-furrow planting patterns of potato on soil evaporation in semiarid area. Chinese Journal of Applied Ecology 18(4):795–800. (in Chinese with English abstract)
217. Tian, Y., Su, D.R., Li, F.M., Li, X.R. 2003. Effect of rainwater harvesting with ridge and furrow on yield of potato in semiarid areas. Field Crops Research 84: 385–391.
218. Wan, W.F., Mei, L., Hong, M., Li, F., Liu, X.D., Gao, X., Zhao, B. 2016. Characteristics of ammonia volatilization and nitrous oxide emission under drip irrigated potato in north of Yinshan of Inner Mongolia.Journal of Irrigation and Drainage 35(8): 36–41. (in Chinese with English abstract)
219. Wang, B.W. 2013. The rules and regulation of farmland carbon cycle under conservational tillage. PhD Thesis, Shandong Agricultural University, Tai'an, China.
220. Wang, C.Y. 2007. Water and nutrient use efficiency of crop with different cultivation methods in rotation system on semi-dry land farming. Dissertation of Master Degree, Northwest Agriculture & Forestry University, Yangling, China.
221. Wang, F.X., Kang, Y.H., Liu, S.P. 2005a. Patterns of water consumption and requirements of potato under dropping irrigation. Agricultural Research in the Arid Areas 23(1): 9–15. (in Chinese with English abstract)
222. Wang, F.X., Feng, S.Y., Hou, X.Y., Knag, S.Z., Han, J.J. 2009a. Potato growth with and without plastic mulch in two typical regions. Field Crops Research 110: 123–129.
223. Wang, H., Sun, L., Liang, J., Li, G.Y., Shi, Y., Bi, S.T., L I, M.Y. 2014. Effect of ratio of nitrogen basal and top dressing and application time on dry matter accumulation and distribution and tuber yield of potato. Chinese Agricultural Science Bulletin 30(24): 224–230. (in Chinese with English abstract)
224. Wang, H.G., Yu, Z.W., Zhang, Y.L., Wang, D. 2010a. Effects of delayed irrigation at jointing stage and irrigation level on consumption amount and resources of water in wheat and farmland evapotranspiration. Acta Agronomica Sinica 36(7): 1183–1191. (in Chinese with English abstract)
225. Wang, H.H. 2008. Research on function of crop-fruit ecological system in middle-south of Losses Plateau. PhD Thesis, Northwest Agriculture& Forestry University, Shaanxi, China.
226. Wang, H.L., Zhang, X.C., Yu, X. F., Ma, Y.F., Hou, H.Z. 2016a. Effect of using black plastic film as mulch on soil temperature and moisture and potato yield. Acta Ecologica Sinica 36(16): 5215–5226. (in Chinese with English abstract)
227. Wang, J.J., Liu, J., Zhang, R.P., Yu, B.S., Li, J.R., Wang, L., Sun, B. 2012. Effects of matching application

of biogas residues and chemical fertilizers on physical and chemical properties and microorganism of soil. Journal of Anhui Agricultural Science 40(13): 7763–7765. (in Chinese with English abstract)
228. Wang, L.W. 2015a. The coordination mechanism and pattern of greenhouse gas reduction and yield increase in potato field. PhD Thesis, China Agricultural University, Beijing, China.
229. Wang, L.X., Chen, Y.Q., Li, C., Shi, J.T., Tao, Z.Q., Nie, Z.J., Zhang, J.S., Sui, P. 2013b. Effects of different drip irrigation systems on yield and water use efficiency of potato in intercropping system of cotton and potato. Acta Agronomica Sinica 39(10): 1864–1870. (in Chinese with English abstract)
230. Wang, Q., Fan, X.L., Klaus, D., Sattemacher, B. 2007. Study on water-saving and water use efficiency of aerobic rice with mulching in South China. Journal of Irrigation and Drainage 26(4): 89–92. (in Chinese with English abstract)
231. Wang, Q., Zhang, E.H., Li, F.M., Wang, X.L. 2005b. Optimum ratio of ridge to furrow for planting potato in micro-water harvesting system in semiarid areas. Transactions of the CSAE 21(2): 38–41. (in Chinese with English abstract)
232. Wang, S.X., Wang, X.D., He, Y.Q. 2010c. Input-output analysis of potato production-A case study of Dingxi, Gansu. Journal of Anhui Agricultural Science 38(4): 2081–2083. (in Chinese with English abstract)
233. Wang, T., He, W.S., Jiang, H.T., Wang, Y.K., Dang, K.K., Zhao, X.X. 2016b. The effects of nitrogen, phosphorus and potassium application on yield and starch content of potato plants. Soil and Fertilizer Sciences 3: 80–86. (in Chinese with English abstract)
234. Wang, W., Zhang, X. 2015. Effects of different irrigation methods on growth and yield of potato in the sandy area of Yulin. Agricultural Research in the Arid Areas 33(4): 153–159. (in Chinese with English abstract)
235. Wang, X.C., Liu, J. 2001. Effect of drought resistance and water save on rice by plastic mulching. Hubei Agricultural Sciences 1: 8-10 (in Chinese)
236. Wang, X.S. 2015b. Experimental study on water-saving irrigation technology of Jinci rice. Shanxi Hydrotechnics1: 83–85. (in Chinese with English abstract)
237. Wang, X., Li, Z.Y., Ma, W.Q., Zhang, F.S. 2010b. Effects of fertilization on yield increase of wheat in different agro-ecological regions of China. Scientia Agricultura Sinica 43(12):2469–2476. (in Chinese with English abstract)
238. Wang, X.B., Liang, G.Q., Zhou, W., Sun, J.W., Pei, X.X., Xia, W.J. 2009c. Effect of optimized nitrogen application on denitrification losses and N_2O emissions from soil in winter wheat/summer corn rotation system in North China. Plant Nutrition and Soil Science 15(1): 48–54. (in Chinese with English abstract)
239. Wang, Y., Ma, J.Q., Li, H.T. 2009e. Study on the culture technology of potato for drought resistance with mechanization. Inner Mongolia Agricultural Science and Technology 5: 56–57. (in Chinese with English abstract)
240. Wang, Y.H., Meng, M.L., Chen, Y.J., Zhang, J.1, Wang, Z.X., Cui, C.L. 2013a. Effect of different film-covering modes on the yield and soil moisture of dry land tillage potato. Chinese Agricultural Science Bulletin 29(3):147–152. (in Chinese with English abstract)
241. Wang, Y.L., Hua, L.N., Tian, W.L., Mei, X.R., Li, Y.Z., Guo, J.X. 2015a. Effects of different moisture treatment on nitrogen leaching and water and nitrogen use efficiency of greenhouse potato. Crops 6: 99–105. (in Chinese with English abstract)
242. Wang, Y.M., Zhang, Z.Y., Fan, M.S. 2009b. Water use efficiency and water production efficiency of potato (*solanum tuberosum* L.) with under-mulch drip irrigation. China Potato 23(3): 148–151. (in Chinese with English abstract)
243. Wang, Y.Q., Li, Y.C., Peng, Z.P., Wang, C.D., Liu, Y.N. 2015b. Effects of dicyandiamide combined with nitrogen fertilizer on N_2O emission and economic benefit in winter wheat and summer maize rotation system. Chinese Journal of Applied Ecology 26(7): 1999–2006. (in Chinese with English abstract)
244. Wang, Y.Y., Hu, C.C. 2011. Soil greenhouse gas emission in winter wheat/summer maize rotation ecosystem as affected by nitrogen fertilization in the Piedmont Plain of Mount Taihang, China Chinese Journal of Eco-Agriculture 19(5): 1122–1128. (in Chinese with English abstract)
245. Wang, Y.Y., Hu, C.S., Cheng, Y.S., Zhang, Y.M., Ming, H., Yang, P.P. 2009d. Carbon sequestrations

and gas regulations in summer-maize and winter-wheat rotation ecosystem affected by nitrogen fertilization in the Piedmont Plain of Taihang Mountains, China. Journal of Agro-Environment Science 28(7): 1508–1515.
246. Wang, Z.B. 2015c. Risk assessment and low carbon strategies of winter wheat and summer maize production in North China Plain under climate change. PhD Thesis, China Agricultural University, Beijing, China.
247. Wang, Z.M., Wang, P., Lan, L.W., Zhou, D.X. 2003. A water-saving and high-yielding cultivation system for bread wheat in Huang-Huai-Hai area of China. Chinese Agricultural Science Bulletin 19(4): 22–25. (in Chinese with English abstract)
248. Wei, H.H., Wang, S.W., Yang, W.J., Sun, H.N., Yin, L.N., Deng, X.P. 2017. Mata analysis on impact of no-Tillage and subsoiling tillage on spring maize and winter wheat yield and water use efficiency on the Loess Plateau. Scientia Agricultura Sinica50(3): 461–473. (in Chinese with English abstract)
249. Wei, J.F., Song, S.H., Liang, Z.H., Wei, D.P., Wei, Q.Y., Liang, H. 2016. Effect of nitrogen application types on nitrogen utilization efficiency and fate of fertilizer for winter-planting potato. Journal of Nuclear Agricultural Sciences 30(1): 178–183. (in Chinese with English abstract)
250. Wei, X.M. 2010. Study on technology of water-saving and yield-increasing of rice in cold field. Dissertation of Master Degree, Northeast Agricultural University, Harbin, China.
251. Wen, D.Z. 2015. Effects of different fertilization on dry plateau wheat yield and water fertilizer use in southern Shanxi. Dissertation of Master Degree, Shanxi Agricultural University, Taigu, China.
252. Wen, R.Y., Guo, Y.D., Zheng, L.S., Jiang, Q,G., Fan, L.S. 2016. Effect of planting density and nitrogen application rate on yield of potato in potato/maize intercropping mode. Journal of Shanxi Agricultural Sciences 44(7): 981–983. (in Chinese with English abstract)
253. Wu, B.F., Yuan, Q.Z., Yan, C.Z., Wang, Z.M., Yu, X.F., Li, A.M., Ma, R.H., Huang, J.L., Chen, J.S., Chang, C., Liu, C.L., Zhang, L., Li, X.S., Zeng, Y., Bao, A.M. 2014. Land Cover Changes of China from 2000 to 2010. Quaternary Sciences 34(4): 723–731. (in Chinese with English abstract)
254. Wu, N. 2014. Current situation and development countermeasures of mechanization production of potato in Zhoucheng county. Farm Machinery 15: 126–127. (in Chinese)
255. Wu, P.P. 2008. Ammonia volatilization and nitrous oxide emission from double rice system in red paddy soil under different fertilizer systems. Dissertation of Master Degree, Nanjing Agricultural University, Nanjing, China.
256. Xia, F.Q., Jiang, X.F., Dong, B., Guo, T.W. 2014. Effects of mulching time and methods on soil hydrothermal status and potato yield on rain-fed field. Journal of Nuclear Agricultural Sciences28(7): 1327–1333. (in Chinese with English abstract)
257. Xiao, X., Zhu, W., Yang, L.L., Deng, Y.P., Huang, L., Zhao, Y.W., Wang, J.F. 2012. Effects of irrigation model and nitrogen fertilization on rice evapotranspiration and yield. Journal of Nanjing Agricultural University 35(4): 27–31. (in Chinese with English abstract)
258. Xiao, Q. 2014. Effects of fertilizer application on potato growth, water use efficiency and soil nutrient content. Dissertation of Master Degree, Inner Mongolia Agricultural University, Inner Mongolia, China.
259. Xiao, X., Zhao, Y.W., Hu, F., Qian, G.M. 2006. Study on function of different water-saving rice model ecosystems in southern China seasonal drought hilly region. Journal of Soil and Water Conservation 20(3): 74–78. (in Chinese with English abstract)
260. Xie, X.L., Qing, X.G., Zhou, J., Wang, K.R., Ma, G.H. 2001. Balance of water in rice paddy and irrigation rate in central China. Research of Agricultural Modernization 22(2): 103–106. (in Chinese with English abstract)
261. Xing, H.F., Shi, X.H., Yang, H.Y., Fan, M.S. 2015. Increase effect of phosphorus absorption of potato population and utilization efficiency by multiple application of phosphate fertilizer with drip irrigation. Journal of Plant Nutrition and Fertilizer 21(4):987–992. (in Chinese with English abstract)
262. Xiong, Z.Q., Xing, G.X., Tsuruta, H., Sheng, G.Y., Shi, S.L., Du, L.J. 2002. Field study on nitrous oxide emission from upland cropping system in China. Soil Science and Plant Nutrition 48(4): 539–546.
263. Xu, X.R., Luo, K., Zhou, B.K., Wang, J.K., Zhang, W.J., Xu, M.G. 2015. Distribution and enrichment characteristics of organic carbon and total nitrogen in moll soils under long-term fertilization.

Chinese Journal of Applied Ecology 26(7): 1961–1968. (in Chinese with English abstract)

264. Xu, Y.M., Liu, H., Dilixiati, Yilihamu, Luo, X.H.,Meng, F.X., Ma, X.W. 2011. Effects of irrigation and nitrogen on flag leaf SPAD, net photosynthetic rate and the yield of winter wheat in the freshly plowed soil of Ili River basin, Xinjiang. Xinjiang Agricultural Sciences 48(12): 2273–2280. (in Chinese with English abstract)

265. Xue, G.Y. 2013. Cultivation techniques for high-yielding and high nutrient use efficiency in rice and their physiological bases. PhD Thesis, Yangzhou University, Yangzhou, China.

266. Xue, J.F. 2015. Effects of tillage on soil carbon and nitrogen in double paddy cropping system of Southern China. PhD Thesis, China Agricultural University, Beijing, China.

267. Xue, J.S., Wei, L.H., Zhang, S.X. 2003. Analysis of the benefit of mechanized operation in potato production. Mechanization In Rural & Pastoral Areas 4: 27–29. (in Chinese)

268. Xue, J.W., Ren, W.J., Yan, C.R. 2014. Effects of plastic film mulching and ridge planting on yield and water use efficiency of potato in Loess Plateau. Chinese Journal of Agrometeorology 35(1): 74–79. (in Chinese with English abstract)

269. Yan, G.X., Zheng, X.H., Cui, F., Yao, Z.S., Zhou, J.W., Deng, J., Xu, Y. 2013. Two-year simultaneous records of N_2O and NO fluxes from a farmed cropland in the northern China plain with a reduced nitrogen addition rate by one-third. Agriculture Ecosystems and Environment 178: 39–50.

270. Yan, M. 2015. Quantitative evaluation of carbon footprint and fertilizer nitrogen fate in agricultural production. PhD Thesis, Nanjing Agricultural University, Nanjing, China.

271. Yang, D.H. 2012. Effect of different fertilization rates and fertilization methods on yields, nutrients, accumulation and tuber quality of potato in Xiangyang. Dissertation of Master Degree, Huazhong Agricultural University, Wuhan, China.

272. Yang, L.H., Meng, M.L., Chen, Y.J., Xiao, Q., Song, S.H. Chen, F. 2013. Effects of fertilizers application on yield and quality of potato. Chinese Agricultural Science Bulletin 29(12):136–140. (in Chinese with English abstract)

273. Yang, L.J., Hao, J.M., Sarengaowa, Yu, C.J., Ji, K. 2013. A new planting methods of potato mechanized furrow ridge in dryland in Guyang. Journal of Chinese Agricultural Mechanization 34(2): 73–76. (in Chinese with English abstract)

274. Yang, M. 2011. The study of key technology for potato high yield in Leishan, Guizhou, China. Dissertation of Master Degree, China Academy of Agricultural Sciences, Guizhou, China.

275. Yang, X., Shang, Q.Y., Wu, J., Liu, Q., Shen, Q.R., Guo, S.W., Xiong, Z.Q. 2010. Methane emissions from double rice agriculture under long-term fertilizing systems in Hunan, China. Agriculture, Ecosystems and Environment 137: 308–316.

276. Yang, X.L. 2015a. Effects of diversified crop rotations on conserving groundwater resource and lowering carbon footprint in the North China Plain. PhD Thesis, China Agricultural University, Beijing, China.

277. Yang, J.J. 2013a. Effects of different fertilization on potatoes and soil quality in the middle arid region. Dissertation of Master Degree, Ningxia University, Ningxia, China.

278. Yang, Y. 2013b. The greenhouse effect, the carbon sink effect and cost-benefit assessments of the Taihu Lake Basin rice cultivation system. Dissertation of Master Degree, Nanjing University, Nanjing, China.

279. Yang, Y.H., Wu, J.C., Li, X.J., Pan, X.Y., Li, Z.J., He, F. 2014. Impact of tillage and soil moisture conservation measures on photosynthetic characteristics and water use of winter wheat. Chinese Journal of Eco-Agriculture 22(5): 534–542. (in Chinese with English abstract)

280. Yang, Z.P. 2015b. Integrated plant nutrient management for white-maize rotation system in Shanxi. PhD Thesis of Shanxi University, Shanxi, China.

281. Yao, F.X. 2011. Studies on physiological mechanism of rice growth and water- and nitrogen- use efficiency under different water and nitrogen regimes. PhD Thesis, Huazhong Agricultural University, Wuhan, China.

282. Yi, Q., Tang, S.H., Pang, Y.W., Huang, X., Huang, Q,Y., Li, P., Fu, H.T., Yang, S.H. 2014. Emissions of CH_4 and N_2O from paddy soil in South China under different fertilization patterns. Journal of Agro-Environment Science 33(12): 2478–2484.(in Chinese with English abstract)

283. Yin, H.F. 2012. Effect of different water and nitrogen conditions on growth and nitrogen leaching of

rice. Dissertation of Master Degree, Nanjing Agricultural University, Nanjing, China.
284. Yin, M., Wang, G.B., Hong, L.F., Fu, L.B., Chen, H., Chen, J.F., Ren, S.S., Zhang, Q.B., Huang, H., Su, F. 2015a. Effect of phosphorus, potassium levels on yield and nutrition absorption of upland potato in Yunnan. Agricultural Research in the Arid Areas 33(1): 91–97. (in Chinese with English abstract)
285. Yin, Z.Y., Pan, Z,C., Jin, S.B., Sui, Q.J. 2015b. The semi-mechanized production technique of spring G1 detoxification variety of potato grown in Yunnan province. Yunnan Nongye Keji 6: 36–38 (in Chinese)
286. Yu, A.Z., Huang, G.B., Cai, Q. 2012. Effect of different tillage treatments on soil respiration of winter wheat farmland in oasis irrigated area Northwest China. Acta Pataculturae Sinica 21(1): 273–278. (in Chinese with English abstract)
287. Yu, X.F., Zhang, X.C., Wang, H.L., Ma, Y.F., Hou, H.Z., Fang, Y.J. 2016. Effects of fertilizer application on water consumption characteristics and yield of potato cultured under ridge-furrow and whole filed plastic mulching in rain-fed area. Chinese Journal of Applied Ecology 27(3): 883–890. (in Chinese with English abstract)
288. Yu, X.R., Li, Y., Shen, X.S., Pan, J.F., Xiao, Y., Zou, H.Z. 2015. Benefit analysis of new planting pattern of three crops per year: "rice-onion-potato" with high efficiency in Anning River valley. Chinese Agricultural Science Bulletin 31(24): 132–136. (in Chinese with English Abstract)
289. Yuan, Y., Liu,C.H., Dai, X.Q., Wang, H.M. 2014. Effects of land-use conversion from double rice cropping to vegetables on CO_2 and CH_4 fluxes in southern China. Chinese Journal of Applied Ecology 26(1): 147–154. (in Chinese with English abstract)
290. Yue, H.L., Zhang, S., Meng, M.L., Wang, L.L., Fan, X.Q. 2013. Effect of phosphorus application amount on product quality and phosphorus fertilizer use efficiency of coated drip irrigation potato. Journal of Inner Mongolia Agricultural University 34(3): 40–45. (in Chinese with English abstract)
291. Zeng, G.J., Qiu, Z.J., Zhang, F.J., Ma, F., Yao, Y.B., Zhang, Q., Wang, R.Y. 2015. Influence of increased temperature on the potato yield and quality in a semiarid district of Northwest China. Acta Ecologica Sinica35(3):830–836. (in Chinese with English abstract)
292. Zeng, X.F., Zhao, S.W., Li, X.X., Li, T., Liu, J. 2012. Main crops carbon footprint in Pingluo county of the Ningxia Hui Autonomous region. Bulletin of Soil and Water Conservation 32(5): 61–65. (in Chinese with English abstract)
293. Zeng, X.F. 2013. The northwest arid areas county farmland ecosystem carbon footprint dynamic. Dissertation of Master Degree, The University of Chinese Academy of Sciences, Beijing, China.
294. Zhai, F.Y., He, Y.N., Ma, G.S., Li, J.P., Wang, Z.H., Hu, Y.S., Zhao, L.Y., Cui, Z.H., Li, Y., Yang, X.G. 2005. Study on the current status and trend of food consumption among Chinese population (Chinese with English abstract). China Journal of Epidemiology 26(7): 485–488.
295. Zhan, Q.H., Chen, J. 2006. Effect of irrigation on yield and water and fertilizer use efficiencies of wheat in Huaibei area. Acta Pedologica Sinica 43(6): 1047–1051. (in Chinese with English abstract)
296. Zhang, C.C., Jiang, R.F., Zhang, F.S., Wang, X.R. 2005. Effect of different N, P_2O_5, K_2O fertilization rate and ratio on nutrients status and tuber yield of potato. Chinese Agricultural Science Bulletin 21(9): 279–283. (in Chinese with English abstract)
297. Zhang, C.W., Dong, B., Guo, T.W., Zhang, T.W., Zhang, G.H., Chen, G.R. 2011a. Effect of supplementary irrigation on potato yield and water use efficiency in semi-arid area of Gansu province. Bulletin of Soil and Water Conservation 31(5): 49–53. (in Chinese with English abstract)
298. Zhang, H. 2017. Cultivation and nitrogen application affect yield and water use efficiency of winter wheat in Qingyang of Gansu province. Journal of Irrigation and Drainage 36(10): 1–6. (in Chinese with English abstract)
299. Zhang, H., Jiang, X.L., Zheng, J.G. 2012a. Studies on water consumption of paddy field in seasonal drought hilly region of Sichuan province. Hybird Rice 27(1): 71–74. (in Chinese with English abstract)
300. Zhang, J., Meng, M.L., Wang, Y.H., Wang, Z.X., Yang, Y.X., Li, X.J. 2012b. The effects of nitrogen phosphorus potassium fertilizer combination on the yield and quality of potato. Crops 4: 124–127. (in Chinese with English abstract)
301. Zhang, J.K. 2011. The emissions of CO_2 and N_2O from purple paddy farm land ecosystem under different tillage systems. Master Dissertation of Southwest University, Chongqing, China.
302. Zhang, L., Liu, J.H., Xu, S.T., Li, Q., Mi, J.Z., Liu, B.H. 2013a. Effect of plant growth nutrients solution

on potato photosynthetic characteristics and yield under different irrigation treatments. Journal of Northwest Agriculture & Forestry University (Nat. Sci. Ed.) 41(2): 145–151. (in Chinese with English abstract)

303. Zhang, M., Ma, Z.X., Xu, Y.K., Zhang, R.H., Gou, J.L., Chen, L., Zhou, R.R., Liu, L.L., He, G.F. 2017. Responses of biological effects and soil fertility to different slow release fertilizers for spring potato in Guizhou. Journal of Southern Agriculture 48(5): 793–799. (in Chinese with English abstract)

304. Zhang, M.Q., Yao, B.Q., Li, J., Kong, Q.B., Chen, Y.H. 2012c. Nitrogen, phosphorus and potassium fertilization for winter potatoes in Fujian. Fujian Journal of Agricultural Sciences 27(9): 982–988. (in Chinese with English abstract)

305. Zhang, M.Z., Niu, W.Q., Lu, Z.G., Wang, J.W., Qiu, X.Q., Li, Y. 2018. The winter wheat's yield and irrigation water use efficiency was increased by moisture irrigation. Journal of Irrigation and Drainage 37(1): Adoi: 10.13522/j.cnki.ggps.2017.0X.016. (in Chinese with English abstract)

306. Zhang, P.L., Guo, T.W., Li, J.F., Zhang, X.C., Guo, X.S. 2013b. Effect of straw covering on yield and water use efficiency of potato and residual effect of N fertilizer in ridges whole film rainfall harvest in no-tillage cultivation. Acta Agriculturae Boreail-occidentalis Sinica 22(3): 93–97. (in Chinese with English abstract)

307. Zhang, Q.P., Yang, X.G., Yang, J., Wang, H.Q., Wang, P., Wang, Z.M., Bouwman. B.A.M. 2005. Photosynthesis characteristics and water use efficiency of aerobic rice under different irrigation treatments. Agricultural Research in the Arid Areas 23(6): 67–72. (in Chinese with English abstract)

308. Zhang, R.P. 2006. Effect of different irrigation methods on growth and development characteristics, yield and water use efficiency in paddy rice. Dissertation of Master Degree, Sichuan Agricultural University, Wuhan, China.

309. Zhang, S.Q., Fang, B.T., Zhang, Y.H., Zhou, S.L., Wang, Z.M. 2009c. Utilization of water and nitrogen and yield formation under three limited irrigation schedules in winter wheat. Acta Agronomica Sinica 35(11): 2045─2054. (in Chinese with English abstract)

310. Zhang, W., Yang, D.H., Huang, X.J., Lou, L.H., Lin, Y.M., Lu, M.X., Zhao, Z.Q. 2016c. Effect of nitrogen rate on the yield, quality and economic benefit of potato in Xiangyang city. Soil and Fertilizer Sciences 1: 72–76. (in Chinese with English abstract)

311. Zhang, W.F., Cao, G.X., Li, X.L., Zhang, H.Y., Wang, C., Liu, Q.Q., Chen, X.P., Cui, Z.L., Shen, J.B., Jiang, R.F., Miao, Y.X., Zhang, F.S., Dou, Z.X. 2016b. Closing yield gaps in China by empowering smallholder farmers. Nature 0: 1–4.

312. Zhang, X.Y., Pei, D., You, M.Z. 2001. Field studies on optimum irrigation scheduling for winter wheat in the piedmont of Mt. Taihang. Shuili Xuebao 1: 90–95.

313. Zhang, X.Y., Liu, X.J., Chen, S.Y., Sun, H.Y., Shao, L.W., Niu, J.F. 2016a. Efficient utilization of various water sources in farmlands in the low plain nearby Bohai Sea. Chinese Journal of Eco-Agriculture 24(8): 995–1004. (in Chinese with English abstract)

314. Zhang, Y., Mu, Y., Zhou, Y., Liu, J., Zhang, C. 2014. Nitrous oxide emissions from maize-wheat field during 4 successive years in the North China Plain. Biogeosciences 11: 1717–1726.

315. Zhang, Y., Zhao, S.W., Liang, X.F., Hua, J. 2009a. Effects of super absorbent on growth of potato and soil water utilization in the mountain area of Southern Ningxia. Agricultural Research in the Arid Areas 27(3): 27–32. (in Chinese with English abstract)

316. Zhang, Y.F., Chen, L.G., Zhu, P.P., Zhang, C.S., Sheng, Q., Wang, Z.C., Zheng, J.C. 2012d. Preliminary study on effect of straw incorporation on net global warming potential in high production rice-wheat double cropping systems. Journal of Agro-Environment Science 31(8): 1647–1653. (in Chinese with English abstract)

317. Zhang, Y.F., Zhou, W., Chen, L.G., Wang, Z.C., Zhu, P.P., Sheng, J., Zheng, J.C. 2013d. Methane and nitrous oxide emission under different paddy-upland crop rotation systems during rice growth season in Taihu Lake region. Chinese Journal of Eco-Agriculture 21(3): 290─296. (in Chinese with English abstract)

318. Zhang, Y.H., Wang, J. 2012. Recycling agricultural carbon footprint calculation in Handan city. Journal of Hebei Agricultural Sciences. 16(12): 60–64. (in Chinese with English abstract)

319. Zhang, Y.T., Zhang, J.Z., Xu, X.C., Peng, C.R., Liu, G.R., Liu, H.B., Liu, W.D. 2011b. Study on the best water and fertilizer management mode for double rice systems in Poyang Lake. China Rice 17(2):

13–16. (in Chinese)
320. Zhang, Z.J., Weng, D.H., Xie, X.Y., Chen, X.Y., Guo, Y.C. 2009b. Characteristics of growth and development and performance of yield and quality for winter-planting potato in different cultivation models. Research of Agricultural Modernization 30(5): 628–632. (in Chinese with English abstract)
321. Zhang, Z.W., Liang, B., Li, J.L., He, H., Jin, S.A. 2013c. Effects of different fertigation methods on yield and nutrient uptake of potato. Chinese Agricultural Science Bulletin 29(38): 268–272. (in Chinese with English abstract)
322. Zhao, C.Q., Wang, S.H. 2017. Study on the cost benefit of potato in Shandong province under the background of potato staple food. Xinjiang State Farms Economy 3: 70–74. (in Chinese)
323. Zhao, H.B., Wang, Z.H., Gao, Y.J., Zhang, W.F. 2016. Investigation and evaluation of household wheat fertilizer application in Shaanxi Province. Journal of Plant Nutrition and Fertilizer 22(1): 245–253. (in Chinese with English abstract)
324. Zhao, J.F. 2004. Water requirement and consumption characteristics and water use efficiency of aerobic rice under different irrigation treatments. PhD Thesis, China Agricultural University, Beijing, China.
325. Zhao, J.S., Yang, Z.X., Ren, Y.P., He, J.L. 2014. Studied on cultivation techniques with water-saving and drip irrigation of potato in arid area of central Ningxia. Liaoning Agricultural Sciences 3: 42–45. (in Chinese with English abstract)
326. Zhao, P.Y., Tuo, D.B., Duan, Y., Gong, Q., Li, H.C., Yu, C.B. 2005. Studies on suitable panting density and time of sowing of potato on dry land in Houshan Area, Inner Monglia Acta Agriculturae Boreali-Sincia20: 10–14. (in Chinese with English abstract)
327. Zhao, R.F., Chen, X.P., Zhang, F.S., Zhang, H.L., Schroder, J., Römheld, V. 2006. Fertilization and nitrogen balance in a wheat–maize rotation system in North China. Agronomy Journal 98:938–945.
328. Zhao, R.F., Chen, X.P., Zhang, F.S. 2009. Nitrogen cycling and balance in winter wheat-summer maize rotation system on North China Plain. Acta Pedologica Sinica 46(4): 684–697. (in Chinese with English abstract)
329. Zhao, Y., Guo, X.N., Zhang, H.B., Wang, Z.H., Zhou, T. 2013. Evaluation and present situation of fertilization in potato in southern mountain region of Ningxia. Soil and Fertilizer 27(5): 281–287. (in Chinese with English abstract)
330. Zhao, Y.W., Xiao, X., Hu, F. 2007. Water utilization efficiency, yield and quality of rice under condition of water-saving in the seasonal dry hilly regions of Jiangxi Province. Agricultural Research in the Arid Areas 25(6): 45–51. (in Chinese with English abstract)
331. Zheng, C.Y., Cui, S.M., Wang, D., Yu, Z.W., Zhang, Y.L., Shi, Y. 2011. Effects of soil tillage practice on dry matter production and water use efficiency in wheat. Acta Agronomica Sinica 37(8): 1432–1440. (in Chinese with English abstract)
332. Zhou, H.L., Mai, Z.Z., Wang, X.J., Yuan, P.C., Jiang, R.L. 2011. Study on special NPK compound fertilizer effect on potato. Ningxia Journal of Agriculture and Forest Science Technology 52(11):11–13.
333. Zhou, L., Long, G.Q., Tang, L., Zheng, Y. 2017. Analysis on N application rates considering yield and N_2O emission in potato production. Transactions of the CSAE 33(2): 155–161. (in Chinese with English abstract)
334. Zhou, W.L. 2016. Evaluation of nutrients and water use efficiency of different maize wheat cropping systems in the North China Plain. PhD Thesis, China Agricultural University, Beijing, China.
335. Zhou, X.Q. 2010. Current situation and development countermeasures of mechanization production technology in potato in Guizhou. Guizhou Agricultural Sciences 38(1): 198–201. (in Chinese with English abstract)
336. Zhu, S.J. 2012. Experiment for water-saving and greenhouse effect of irrigation mode in cold rice area. PhD Thesis, Northeast Agricultural University, Harbin, China. (in Chinese)
337. Zhuang, D.X. 2015. Influence of the different cultivation and irrigation pattern on rice water requirement rule and yield. Dissertation of Master Degree, Northeast Agricultural University, Harbin, China.
